# Multifunctional Polymer Matrix at the Buried Interface Boosting Stability and Efficiency in Perovskite Solar Cells

**DOI:** 10.1002/smll.202507718

**Published:** 2025-09-27

**Authors:** Huiming Luo, Zhijie Gao, Himal Muwanwella, Galyam Sanfo, Ying Lu, Shurui Yang, Yinheng Ren, Shanyue Hou, Xiang Liu, Preetam Kumar Sharma, Robert Palgrave, Muhammad Tariq Sajjad, Peng Huang, Mojtaba Abdi‐Jalebi

**Affiliations:** ^1^ Institute for Materials Discovery University College London Malet Place London WC1E 7JE UK; ^2^ Key Laboratory of Advanced Technologies of Materials Ministry of Education School of Materials Science and Engineering Southwest Jiaotong University Chengdu 610031 P. R. China; ^3^ Research Institute of Frontier Science Southwest Jiaotong University Chengdu 610031 P. R. China; ^4^ School of Engineering and Design London South Bank University 103 Borough Road London SE1 0AA UK; ^5^ Department of Chemistry University College London London WC1H 0AJ UK; ^6^ School of Materials Science and Engineering Beihang University Beijing 100084 P. R. China

**Keywords:** perovskite solar cells, polymer matrix, stability, steric effect, tin oxide nanoparticles

## Abstract

To drive further advancements, increasing attention has been directed toward optimizing the buried interface in perovskite solar cells, which not only influences carrier accumulation and recombination but also plays a decisive role in the overall quality of deposited perovskite thin films. In this work, sodium hyaluronate is employed to disperse the SnO_2_ nanoparticles and facilitate the formation of uniform and compact SnO_2_ films. The polymer matrix endows a homogeneously dispersed SnO_2_ precursor with long‐term stability via steric effect and electrostatic repulsion. This modification effectively eliminates the oxygen vacancies and dangling hydroxyl bonds at the interface. The buried interface is modulated and, in turn, oriented perovskite grains, relaxed residual strain, and well‐matched energy alignment enable superior enhancements for obtained devices. Thus, the target device exhibits a champion power conversion efficiency of 25.11% with negligible hysteresis, compared to the control (24.45%). The unencapsulated device still maintains 90% of its original efficiency after being stored at ambient air (humidity >50%) for 1000 h. Hence, this strategy provides a promising approach for enhancing the intrinsic stability of both SnO_2_ and perovskite layers, marking a step forward toward the commercialization.

## Graphical abstract

Sodium hyaluronate (SHA) serves as a polymer matrix that stabilizes the SnO_2_ dispersion and, by reconstructing the buried interface, holistically enhances both the overall performance and long‐term stability of perovskite solar cells. SHA promotes the regular arrangement of SnO_2_ during thin‐film formation and eliminates oxygen defects. It also facilitates the vertical growth of the perovskite grains and reduces void formation.

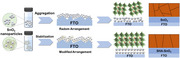

## 1 Introduction

Tin oxide has been widely used in perovskite solar cells (PSCs), as an electron transport layer (ETL), and has made great contributions to promoting the power conversion efficiency (PCE) exceeding 27%.^[^ ^1^, ^2^ ^]^ It presents superior properties as an ETL, such as high transparency, low preparation temperature, excellent electron mobility, solution processible, and excellent stability.^[^ ^3^ ^]^ Since the introduction of SnO_2_ colloidal dispersion in typical planar n–i–p PSCs in 2016, it has witnessed numerous breakthroughs in the evolution of PCEs.^[^ ^4^ ^]^ However, intrinsically unstable SnO_2_ particles tend to agglomerate by van der Waals forces, negatively affecting the electronic properties and surface roughness of the obtained ETLs.^[^ ^5^, ^6^ ^]^ Besides, defects like oxygen vacancies (*V_O_*), and dangling hydroxyl groups (─OH) formed within the bulk and on the surface during the low‐temperature preparation procedure, leading to the charge carrier accumulation and becoming the non‐radiative recombination center.^[^ ^7^ ^]^ Even within the perovskite layer, the quality of bottom buried layer has a significant effect on the crystal nucleation and growth of upper absorbing perovskite layer.^[^ ^8^ ^]^ Except for film formation, lattice mismatch, bandgap offset, and interfacial residual stress hinder the performance of obtained devices, especially for open‐circuit voltage (*V_oc_*) and fill factor (FF). These imperfections limit the interior carrier extraction and transport, consequently reducing the PCE of the devices.

Various feasible strategies have been explored to develop smooth and dense SnO_2_ ETL, aiming at ideal buried interfaces and tackling the problems previously mentioned. Element doping is proven to be a reasonable way to enhance the electrical conductivity of SnO_2_, contributing to the energy band structure and carrier concentrations. Till now, elements like Zn, Al, Li, Nb, Y, and Zr have been utilized as effective dopants to promote the electrical properties of obtained SnO_2_ thin films.^[^ ^9^, ^10^, ^11^, ^12^, ^13^, ^14^ ^]^ Moreover, interface engineering, through incorporation of Lewis acids and bases, alkaline cations, or halogen ions, has been extensively employed to passivate surface defects.^[^ ^15^, ^16^, ^17^ ^]^ As they form new chemical bonds with SnO_2_, targeting at the undercoordinated tin and oxygen atoms. For example, early in 2018, researchers had found that with the incorporation of KCl, both positively charged and negatively charged ionic defects can be passivated, and the hysteresis was largely eliminated.^[^ ^18^ ^]^

Generally, commercial colloidal dispersions are stored under refrigerated conditions to preserve their dispersibility and chemical stability, as thermal energy can promote nanoparticle aggregation and sedimentation. In this case, additive engineering appears to be a more effective approach to addressing the instability of SnO_2_. Specifically, functional molecules like Poly(ethylene glycol) diacrylate, heparin potassium, sodium gluconate, and polyethylene glycol can form a strong bonding with SnO_2_ via van der Waals force.^[^ ^7^, ^19^, ^20^, ^21^, ^22^ ^]^ These molecules not only prevent the aggregation of SnO_2_, but also inhibit the formation of defects during the perovskite film formation. Although these methods can temporarily prevent the agglomeration of tin oxide, the issue of ensuring the long‐term storage stability of SnO_2_ solutions at ambient air still requires further investigation.

In this work, we employ sodium hyaluronate (SHA), a biocompatible polymer, to regulate the arrangement of SnO_2_ colloid suspension. Benefiting from its polymer matrix, it endows a homogeneously dispersed SnO_2_ precursor by creating a steric barrier around the SnO_2_ particles, as well as introducing the electrostatic repulsion among the particles. Consequently, the obtained SnO_2_ thin film exhibits a uniform morphology, stunning wettability, and reduced surface oxygen vacancies. The functional groups in SHA can also anchor the perovskites onto the ETL as a bridge and contribute to well‐arranged perovskite grains, reducing defects and eliminating the voids both in the bulk and at the interface. As a result, the champion device reaches a PCE of 25.11%, with a reduced hysteresis, and the average open‐circuit voltage increased from 1.16 to 1.19 V, due to the improved interface contact. The target device can maintain 90% of its original efficiency after being stored in an ambient environment (≈50% RH) environment over 1000 h without encapsulation. This modified strategy of SnO_2_ provides a holistic approach for enhancing the intrinsic stability of both SnO_2_ and perovskite, which is remarkable for the commercialization of PSCs.

## 2. Results and Discussion

**Figure 1:**
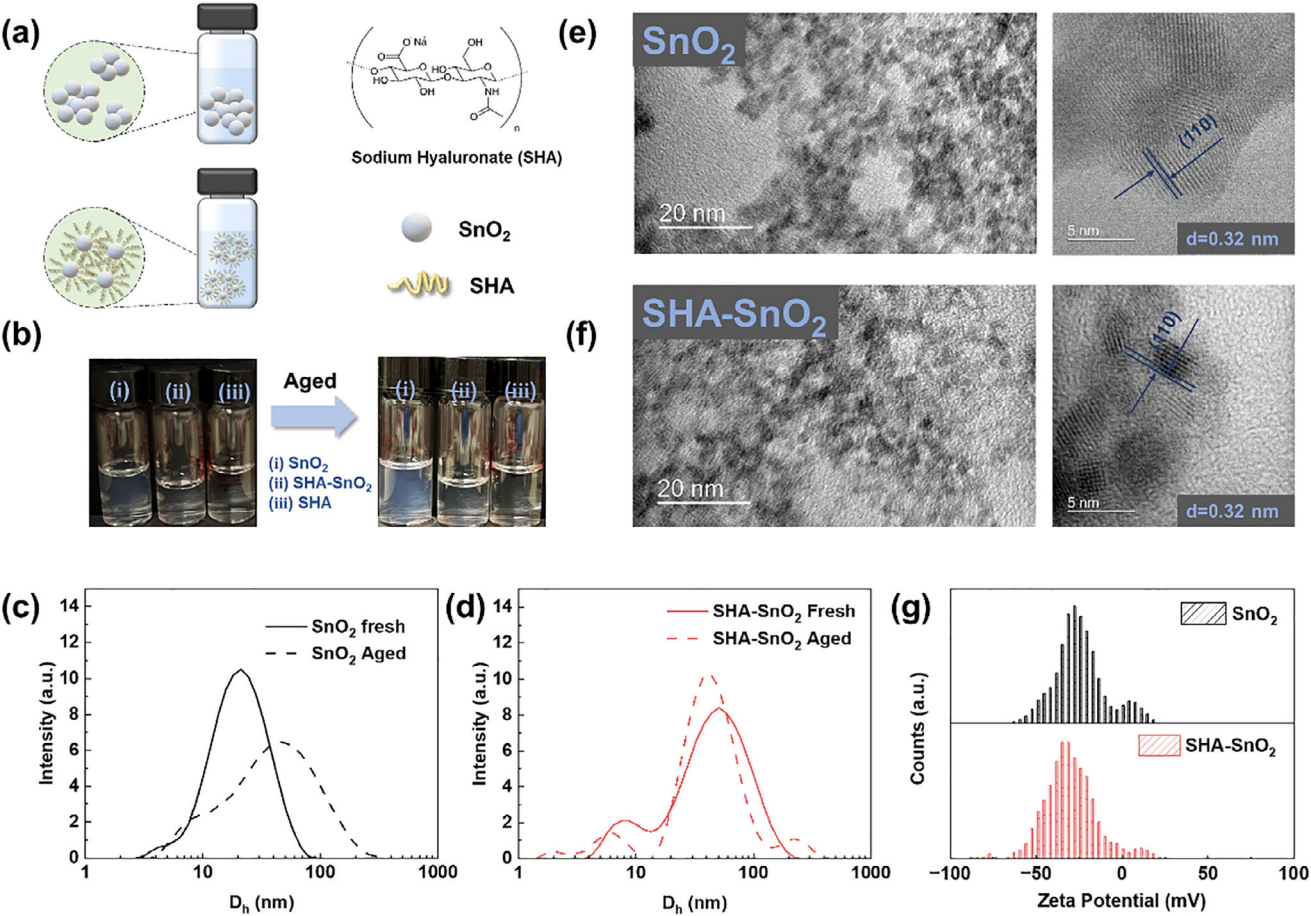
Characterizations on SHA molecule and SnO_2_ nanoparticles in dispersion. a) Schematic of the distribution of SnO_2_ nanoparticles in dispersion and molecule structure of SHA; b) pPhotos of fresh and aged SnO_2_ colloidal dispersions with and without SHA; c,d) DLS spectra of SnO_2_ colloidal dispersion with and without SHA; e,f) TEM images of SnO_2_ nanoparticles with and without SHA; g) zeta potential of SnO_2_ colloidal dispersions with and without SHA.

The molecular structure is shown in **Figure** **1a**. SHA is adsorbed onto SnO_2_ nanoparticles via chelation between the carbonyl oxygen (electron donor) and undercoordinated Sn ions (Lewis acid sites), forming Sn─O─C bonds. The absorption of SHA over SnO_2_ nanoparticles acts as a protective layer, becoming a steric barrier that prevents adjacent SnO_2_ nanoparticles from attracting each other.^[^ ^23^ ^]^ When dissolved in water, the carboxyl group (─COONa) will dissociate into negatively charged ─COO^−^ and Na⁺, stabilizing the nanoparticles via electrostatic forces. Figure 1b presents the photos of SnO_2_ solutions with and without SHA placed at room temperature for a week. SHA can be easily dissolved in aqueous solution and form a transparent mixture. More importantly, the control solution turns blue, cloudy, and hazy with visible precipitate after aging. While the one with SHA remains clear due to the SHA polymer matrix, indicating significant stabilization. To analyze the changes in the solution, dynamic light scattering (DLS) is used to understand the distribution of the SnO_2_ size in water. As shown in Figure 1c,d, a typical Gaussian distribution is observed. For the control SnO_2_, the particle size distribution shows a broad peak ≈20.86 nm, and the dominant peak shifts to 46.58 nm after aging, indicating the formation of SnO_2_ oligomers. With the addition of SHA, the dominant peak increases to 40.62 nm for the fresh, attributed to the absorption of SHA on the surface of SnO_2_, and slightly increases to 49.92 nm for the aged, demonstrating the stabilization of SHA for the long‐term storage of SnO_2_ dispersion. However, due to the effect of multiple scattering and the presence of electrical double layer, DLS tends to overestimate the size.^[^ ^24^ ^]^ Thus, transmission electron microscopy (TEM) is conducted to determine the actual particle size. Both the SnO_2_ and SHA–SnO_2_ nanoparticles are ≈5 nm in size (Figure 1e,f). Furthermore, for SHA–SnO_2_, the particles are uniformly spread with clear boundaries, while the control is witnessed to be stacked. Zeta potential measurement is conducted to prove the effect from electrostatic repulsion. The primary zeta potential (Figure 1g) for pure SnO_2_ aqueous solution is ≈−24.41 mV, and increases to −30.06 mV with the addition of SHA, supporting that the existence of SHA enhances the electrostatic repulsion between particles and prevents the aggregation.

The nanoparticles are deposited onto the FTO substrate by spin‐coating method, followed by an annealing process at 150 °C. As the melting point for SHA is over 200 °C, it will remain in the SnO_2_ layer after annealing. The optical transmittance is measured by UV–Vis spectroscopy, and there is no change found in the range from 400 to 900 nm (Figure S1, Supporting Information), suggesting that the introduction of SHA doesn't block the incident light and photogenerated carrier. According to classic homogeneous nucleation theory, the bottom substrate affects the resulting thin film in two aspects: flatness and uniformity, as well as interfacial contact.^[^ ^25^ ^]^ Scanning electron microscopy (SEM) and atomic force microscopy (AFM) are conducted to study the morphologies of the obtained SnO_2_ and SHA–SnO_2_ thin films. Negligible change in domain size is observed with SHA concentration up to 2 mg mL^−1^ (Figure S2, Supporting Information). Further increasing SHA concentration leads to enlarged and flake‐shaped domains, which may hinder the carrier transport. In contrast to the control thin film, the surface roughness is reduced (Figure S3, Supporting Information), where the root‐mean‐square roughness is 8.93 nm for SnO_2_ and 7.44 nm for SHA–SnO_2_, respectively. A more uniform and compact film is beneficial to further perovskite deposition. SHA–SnO_2_ exhibits a smaller water contact angle (12.60°) in Figure S4, Supporting Information; compared with SnO_2_ (37.29°), suggesting the improved wettability from the introduction of carboxylate and hydroxyl groups of SHA. It is expected that homogenous crystal nucleation and growth will happen during the deposition of perovskite layer.

**Figure 2:**
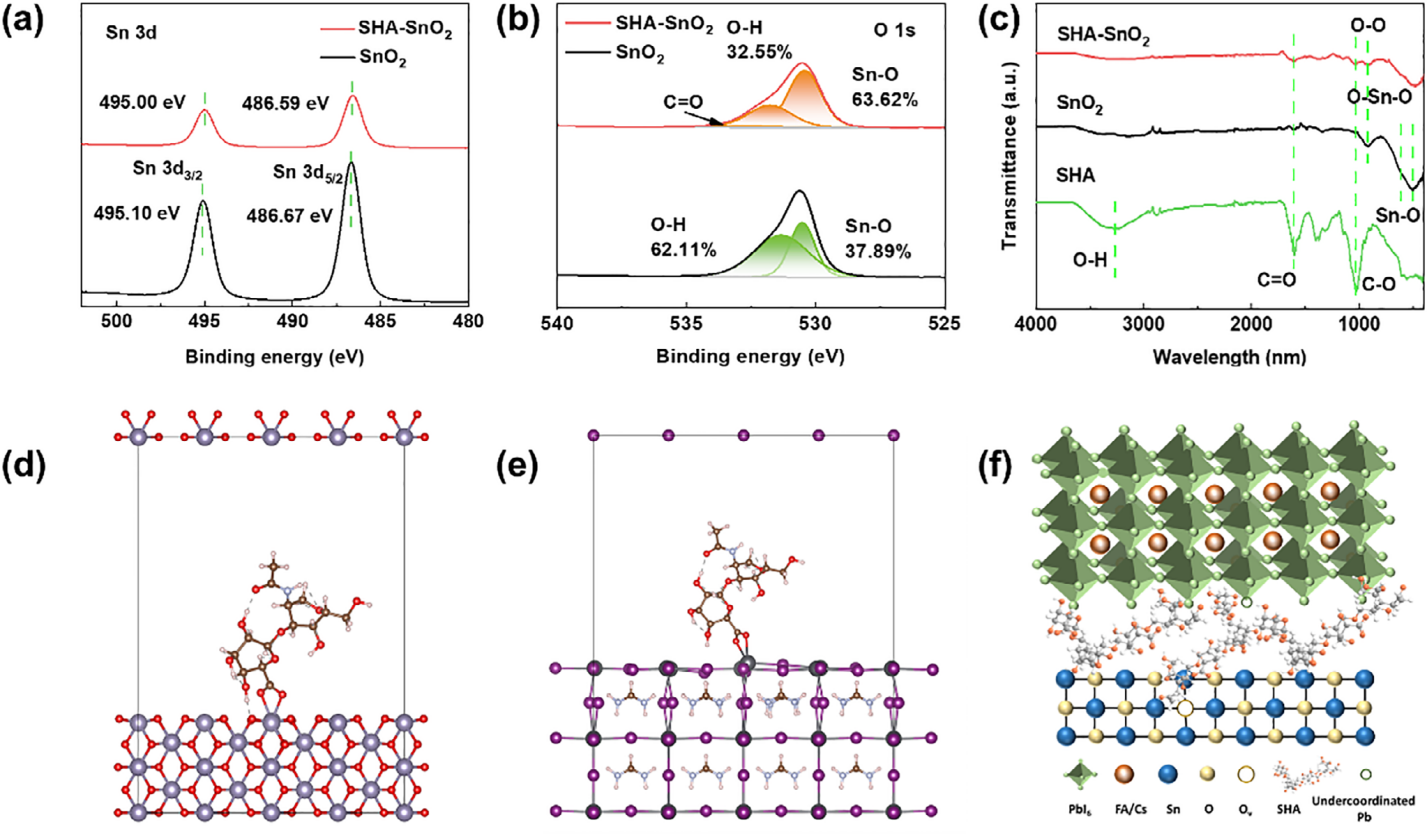
Interactions between SnO_2_ and SHA. a,b) XPS spectra of Sn 3d and O 1s for SnO_2_ and SHA–SnO_2_ thin films; c) FTIR spectra; d,e) adsorption energy of SHA adsorbed onto SnO_2_ surface and perovskite surface; f) schematics of interactions with SHA modification.

To further identify the chemical interaction between SnO_2_ and SHA, the X‐ray photoelectron spectroscopy (XPS) and Fourier‐transform infrared (FTIR) spectroscopy are applied on SHA, SnO_2,_ and SHA–SnO_2_ thin films. For SHA‐modified SnO_2_ thin film, the peak ascribing to Sn 3d shifts to lower binding energy in **Figure** **2**a, indicating the strong interaction between SHA and Sn atom, which increases the surrounding electron density of Sn.^[^ ^26^, ^27^ ^]^ In O 1s spectra of Figure 2b, the peaks located at 530.52 eV are attributed to the lattice oxygen (Sn‐O), while those at 531.34 eV are related to surface hydroxyl group.^[^ ^28^ ^]^ The latter will introduce the trap states and notably contribute to the non‐radiative recombination, thus hindering charge extraction and transfer.^[^ ^29^ ^]^ Here, the ratio of those components is calculated and marked in Figure 2b. In contrast to the control SnO_2_, the ratio of lattice oxygen increases from 37.89% to 63.62% for the modified one. In other words, the oxygen defects, including oxygen vacancy and surface hydroxyl group, are suppressed, which will promote the carrier transport and inhibit the accumulation of carriers and ion migration. Additionally, the peak of C═O can be found at 532.82 eV in SHA–SnO_2_ thin film, confirming the existence of SHA. In the FTIR spectra (Figure 2c), the broad absorption band (3000–3500 cm^−1^) corresponds to the O─H stretching vibration for the pure SHA film. The characteristic peaks at 1020 cm^−1^, 1602 cm^−1^ belong to the C─O and C═O groups, respectively.^[^ ^30^ ^]^ Pure SnO_2_ exhibits typical stretching vibrations of Sn─O at ≈505 cm^−1^, symmetric vibrations of O─Sn─O at 615 cm^−1^ and O─O vibrations at 910 cm^−1^ from the oxygen absorbed at the SnO_2_ surface.^[^ ^31^, ^32^ ^]^ The FTIR spectra of modified SHA–SnO_2_ film contains both the characteristic peaks from SHA and SnO_2_, proving the successful incorporation of SHA. As reported, metal oxide surfaces always have lots of hydroxyl groups due to water absorption and surface oxidation. Those surface hydroxyl groups can be eliminated when connected with the ─OH and ─C═O in SHA via hydrogen bonding.^[^ ^33^, ^34^ ^]^ Meanwhile, it is anticipated that these functional groups can also coordinate the Pb^2+^ and halide ions in the perovskite layer simultaneously, to enhance the quality of deposited perovskite.^[^ ^35^ ^]^

To investigate the interactions of SHA with SnO_2_ and perovskite, density functional theory (DFT) calculations is performed to quantify the adsorption energy (*E_ads_*). The chemical structure is illustrated in Figure S5a (Supporting Information), and the electrostatic potential (ESP) of SHA with or without Na^+^ is presented in Figure S5b,c (Supporting Information). In Figure S5b (Supporting Information), the negatively charged region is mainly located around the O atoms from O─H, CONH, and COO^−^ group. The Na cation creates a strong localized positive region around the COO^−^ group. After dissolving in water, SHA undergoes almost complete deprotonation, forming carboxylate groups. Therefore, the ESP without Na^+^ is also presented in Figure S5c (Supporting Information). The ESP is dominated by strongly negative regions centered on the–COO^−^ of the glucuronic acid residues. These negative potential zones extend broadly and create a relatively uniform “red” in ESP visualizations, with only minor localized positive regions from hydroxyl or amide hydrogens. The negatively charged zone not only can act as Lewis acid to interact with the under‐coordinated Pb^2+^, but also fill the oxygen vacancies in tin oxide, target at the negatively charged defects. As reported in previous work, those molecules prefer to interact with the SnO_2_ and perovskite in the vertically‐stand way via COO^−^ group, in comparison with the N─H or C═O interactions. The adsorption energy of SHA with SnO_2_ and perovskite is calculated when SHA form a perpendicular pose to the exposed surface with COO^‐^ group. Interaction between SHA and SnO_2_ or perovskite is calculated and shown in Figure 2d,e. The adsorption energy between SHA and SnO_2_ or perovskite is −1.86 and −1.18 eV, respectively. It is indicated that SHA is prone to interact with under‐coordinated Sn in SnO_2_ and Pb in perovskite via COO─ groups. The O─H groups can interact with the oxygen in the way of O─H∙∙∙O─Sn, thus reducing the surface dangling bonds. A visualized illustration is shown in Figure 2f, to exhibit all sorts of interactions between SnO_2_ and SHA, as well as those between SHA and perovskite.

**Figure 3:**
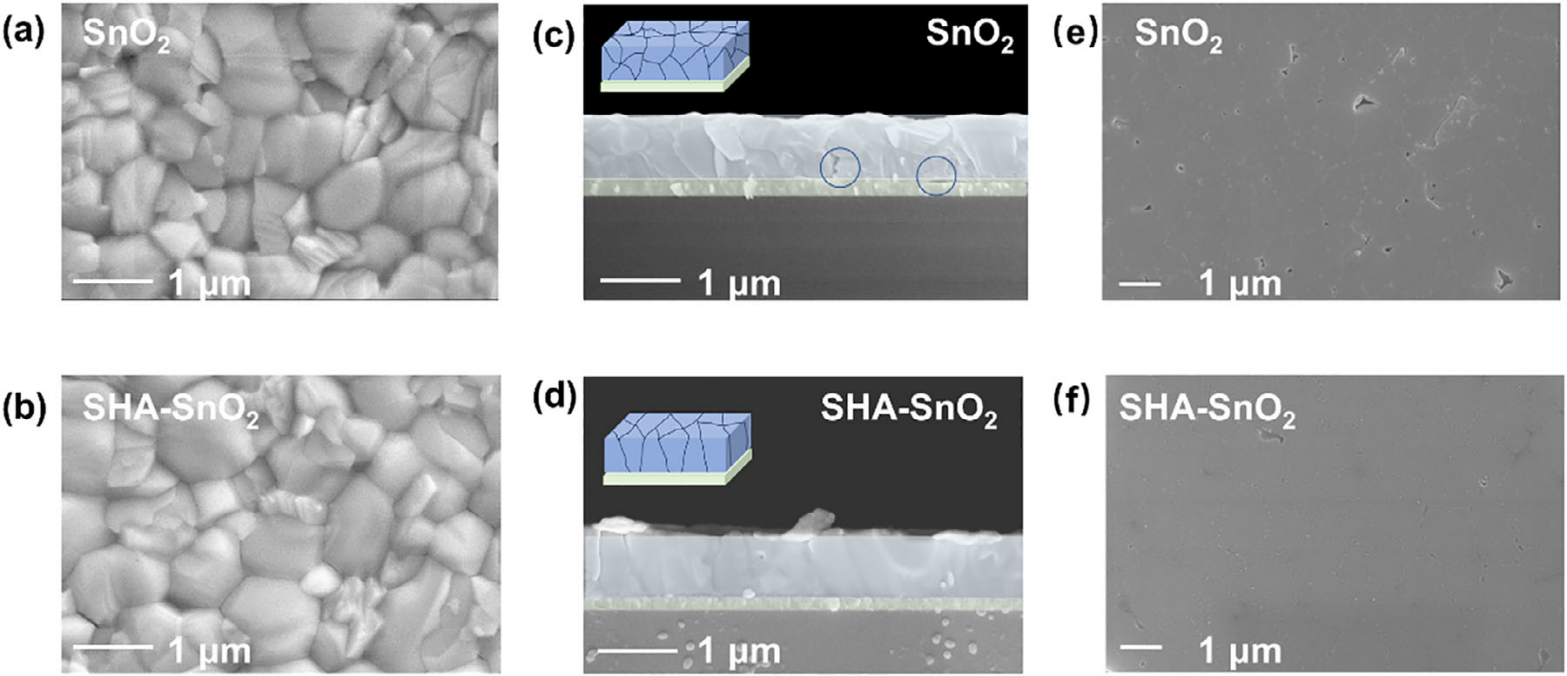
Morphologies of perovskite films. a,b) Top‐view SEM images, c,d) cross‐sectional view SEM images of perovskite films deposited on SnO_2_ and SHA–SnO_2_; inserted: schematic representations of grain arrangement; e,f) SEM images of buried surface of perovskite films after peeled off from substrates.

To investigate the impacts of SHA on the perovskite films, SEM and AFM are first employed to see the morphologies of the obtained perovskite films deposited on SnO_2_ and SHA–SnO_2_ substrates. As exhibited in **Figure** **3**a,b, the perovskite films are fully covered on the substrates. Notably, with the incorporation of SHA, the dominant grain size remarkably increases from 700 to 1000 nm as summarized in Figure S6 (Supporting Information). The formation of more uniform, denser, and larger grains, and along with significantly reduced grain boundaries are attributed to the reduced Gibbs free energy at the substrates, which aligns with the lower contact angle for SHA–SnO_2_.^[^ ^36^ ^]^ AFM images also reveal a flatter and smoother surface with RMS reduced from 29.10 to 24.10 nm (Figure S7, Supporting Information). Moreover, cross‐sectional SEM images in Figure 3c,d reveal the buried interface is also optimized. Control films display several voids at the interface and in the bulk, which would act as the carrier traps and accelerate the degradation. Compared with the control film (Figure 3c), the grains of SHA‐modified perovskite film are well‐oriented and vertically arranged (Figure 3d), beneficial for the carrier transport and energy loss. As shown in Figure S8 (Supporting Information), the film is encapsulated with glass via UV‐curing resin and peeled off after solidification. A few voids at the grain boundaries are observed for the control, acting as the nonradiative recombination sites and initializing the degradation. While the bottom surface of perovskite films deposited on SHA–SnO_2_ is smooth and compact. As SHA can interact with the PbI_2_, regulating the nucleation and growth of perovskite.

**Figure 4:**
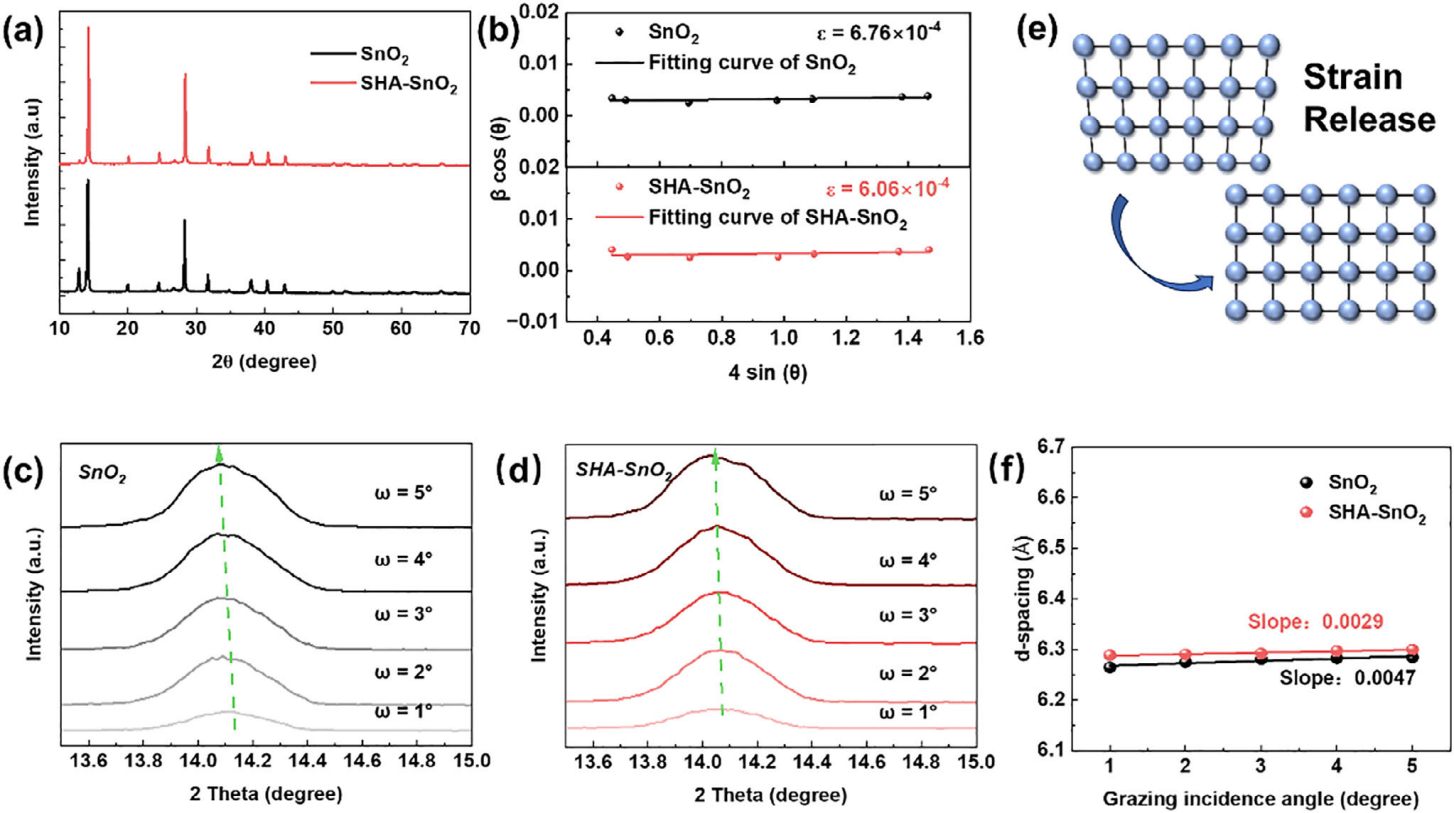
Stain analysis of perovskite thin films. a) XRD, and b) Williamson‐Hall plots of perovskite films deposited on SnO_2_ and SHA–SnO_2_; GIXRD of perovskite films deposited on SnO_2_ c) and SHA–SnO_2_ d) with grazing incidence angle (ω) ranging from 1° to 5°; e) illustration of strain release; f) linear fitting of d‐spacing versus grazing incidence angle.

X‐ray diffraction (XRD) is conducted to study the crystallinity of perovskite thin film. Typical peaks at 14.13° and 28.40° belong to the (001) and (002) crystal planes of perovskite, respectively (**Figure** **4**a).^[^ ^37^ ^]^ The peaks at 12.83° are ascribed to the existence of PbI_2_, leading to the poor carrier transport and inherent instability.^[^ ^38^ ^]^ Compared with the control film, no extra peaks are found after incorporation of SHA, indicating that SHA doesn't for new phase. The stronger intensity of main peaks for SHA–SnO_2_ perovskite thin film and reduced residual PbI_2_ peaks suggest the improved crystallinity, which is in agreement with the superior morphology we observed from SEM. Williamson‐Hall analysis is further conducted to investigate the effects of SHA as buried layer on microstructures, particularly lattice strain. The micro‐strain can be extracted from the following equation:

(1) $$\beta \cos\theta =\varepsilon \left(4sin\theta \right)+\frac{k\lambda }{D}$$

where *β* is the full width at half maximumof XRD peak; *θ* is the diffraction angle; *k* is Scherrer constant; *λ* is X‐ray wavelength (1.5406 Å for Cu Kα radiation); *D* is the crystallite size; and ɛ is the micro‐strain; As shown in Figure 4b, slope is extracted by plotting β*cos*θ versus 4*sin*θ. The incorporation of SHA leads to a released lattice micro‐strain from 6.76 × 10^−4^ to 6.06 × 10^−4^.

To further gain deeper insight into the residual strain, Grazing incidence X‐ray diffraction (GIXRD) measurements are conducted by tuning the grazing incidence angle (ω) from 1° to 5° in Figure 4c,d. Along with the increase of ω, the peak of (001) plane shifts toward lower diffraction angle from 14.13° to 14.08°. Consequently, an increased d spacing is calculated via Bragg equation, indicating the presence of tensile strain. In contrast, the modified thin film shows negligible shift, which suggests the elimination of tensile strains. By plotting the d spacing versus ω, the slope decreases from 0.0047 of control film to 0.0029 of modified film. A visualized strain release process is inserted for an intuitive understanding with the addition of SHA in Figure 4e,f.

Additionally, in Figure S9 (Supporting Information), a slight increased absorption intensity in the range of 600–800 nm is observed. The steady‐state photoluminescence (PL) spectra are shown in Figure S10 (Supporting Information). The PL peaks is quenched for the perovskite deposited on SHA–SnO_2_ film, suggesting more efficient carrier extraction and transfer. Meanwhile, time‐resolved PL (TRPL) is conducted to analyze the carrier extraction and transfer kinetics in Figure S11 (Supporting Information). The TRPL decay can be fitted by a double exponential decay equation, *y(t) = y_0_+A_1_ exp(−t/τ_1_)+A_2_exp(−t/τ_2_)*, where *τ_1_* and *τ_2_* refers to the lifetime of fast and slow decay processes, respectively. Here, in contrast to the pristine perovskite, the *τ_1_* decreases from 73.23 to 46.10 ns, and *τ_2_* decreases from 392.40 to 255.90 ns after addition of SHA. The faster decay means the carrier extraction becomes more effective, which originates from the more uniform ETL and a better contact at buried interface. Moreover, as displayed in Figure S12 (Supporting Information), the peak belonging to Pb 4f_7/2_ orbit shifts from 138.56 to 138.45 eV, and the peak for Pb 4f_5/2_ orbit shifts from 143.39 to 143.29 eV. The change toward lower binding energy indicates the interaction between SHA and undercoordinated Pb^2+^ by ─OH∙∙∙Pb or C─O─C∙∙∙Pb, as those interactions increase the electron cloud density around Pb.^[^ ^39^ ^]^

**Figure 5:**
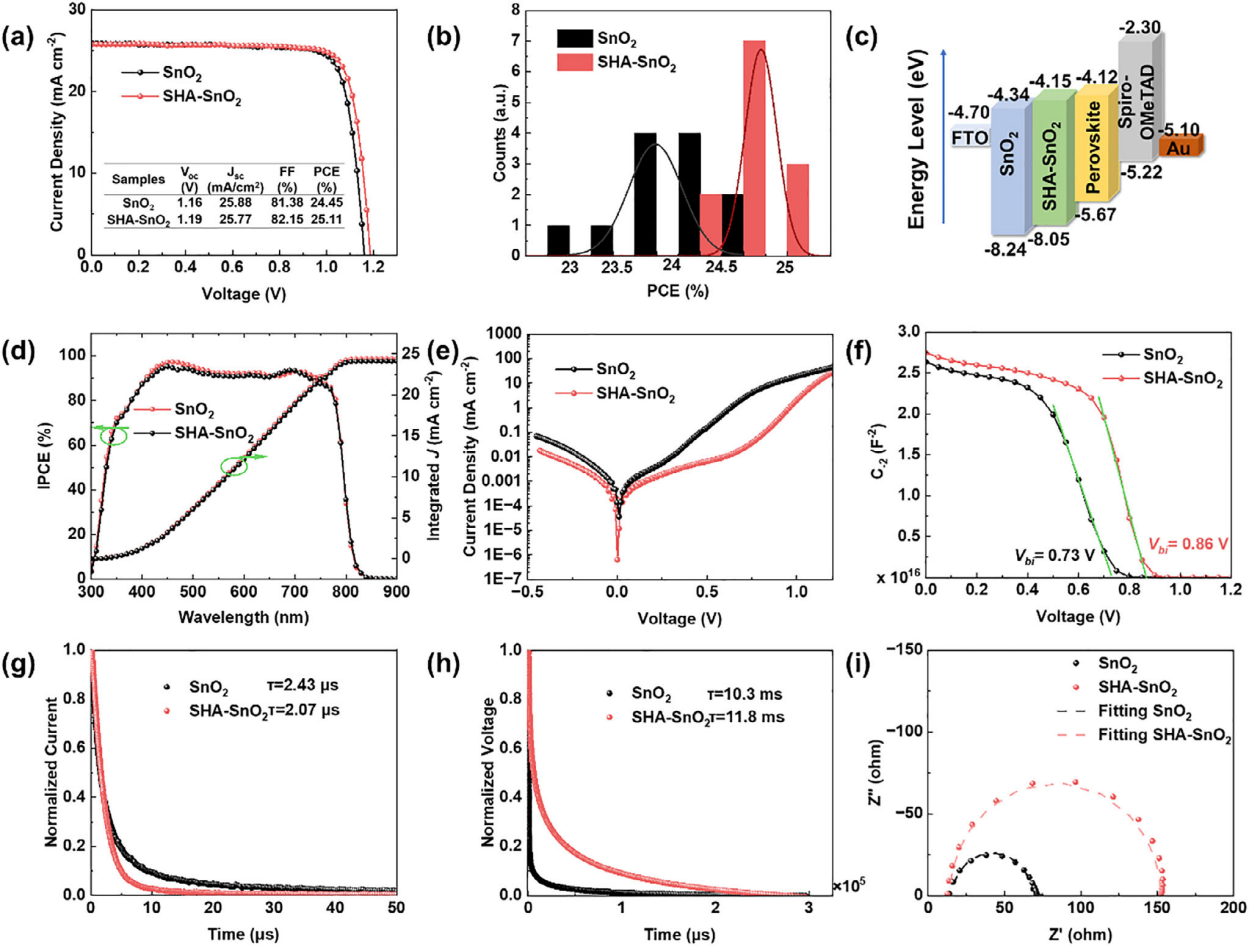
Photovoltaic performances and charge dynamics analysis. a) *J*–*V* curves of champion devices with and without SHA modification at reverse scanning, measured under full simulated solar illumination conditions (AM1.5, 100 mW cm^−2^).; b) PCE distributions of 12 devices with and without SHA; c) energy level diagram of devices; d) IPCE spectra and integrated current density for SnO_2_ and SHA–SnO_2_ PSCs; e) Dark *J*–*V* curves; f) Mott–Schottky plots; g) transient photocurrent and h) transient photovoltage; i) Nyquist plot of EIS measurement.

Thus, the devices based on the structure of FTO/SnO_2_(SHA–SnO_2_)/Perovskite/Spiro‐OMeTAD/Au are fabricated to study the effects of SHA on the photovoltaic performance. First, to determine the best concentration of SHA, 1–4 mg mL^−1^ SHA is added to the SnO_2_ dispersion to prepare the ETL in the devices. The photovoltaic performances in the same batch are summarized in Figure S13 and Table S1 (Supporting Information). Combined with the morphologies of SnO_2_ thin film from SEM images (Figure S2, Supporting Information), the optimal performance is observed at 2 mg mL^−1^. With increase of SHA concentration, the *V_oc_* significantly increases due to the improved interface contact, accelerated carrier extraction, and vertically arranged perovskite grains. And too much SHA may block the carrier transfer. **Figure** **5**a exhibits *J*–*V* curves of the optimal devices at reverse scanning. Notably, the device on SHA–SnO_2_ reaches a remarkable PCE of 25.11%, in contrast with 24.45% of control devices. The *V_oc_* is enhanced from 1.16 to 1.19 V, and*FF* increases from 81.38% to 82.15%. The hysteresis index (HI) is studied by the *J*–*V* curves scanned at forward and reverse direction in Figure S14 and Table S2 (Supporting Information), where the HI is 12% and 9% for the champion SnO_2_ and SHA–SnO_2_ devices, respectively. Statistical analysis of PCE, *J_sc_*, *V_oc,_* and FF are presented in Figure 5b and Figure S15 (Supporting Information). SHA–SnO_2_ devices present a narrower distribution than SnO_2_ devices in the histograms, indicating an excellent reproducibility. The average photovoltaic parameters are listed in Table S2 (Supporting Information) to highlight the superiority of SHA–SnO_2_ PSCs, caused by the enlarged perovskite grains, oriented growth, and reduced trap density. A consistent improvement in *V_oc_* is observed across all devices (Figure S15a, Supporting Information), which may result from the better energy level alignment between ETL and perovskites. Moreover, the average hysteresis is reduced by 6%, indicating the elimination of charge accumulation and superior electronic contact at the interface.

Ultraviolet photoelectron spectroscopy (UPS) is measured to evaluate the energy levels in Figure S16 (Supporting Information). Work function (*WF)* can be calculated from the secondary electron cutoff by the following equation: *WF* = 21.22─*E_cutoff_*, which is 4.43 and 4.32 eV for SnO_2_ and SHA–SnO_2_, respectively. Then, valence band maximum (VBM) can be calculated by the onset edge of UPS spectra. Considering the bandgap, *E_g_* (3.9 eV) of SnO_2_ got from UV–vis spectra, the conduction band minimum (CBM) can be determined accordingly. Therefore, the energy level diagram is illustrated in Figure 5c, showing that the energy barrier between SnO_2_ and perovskite is shortened by SHA.^[^ ^7^ ^]^ The well‐matched energy levels can facilitate the electron transfer, contributing to the increase in *V_oc_*.

Figure 5d presents the incident photon‐to‐electron conversion efficiency (IPCE) spectra and the integrated *J_sc_* of corresponding devices. The integrated *J_sc_* of SHA–SnO_2_ devices is increased from 24.11 to 24.38 mA cm^−2^, which is close to the one extracted from *J*–*V* curves. The dark *J*–*V* curve is depicted in Figure 5e. The leakage current density of SHA‐modified device is smaller than that of control device. As it originates from the poor interfacial contact, the lower dark current reflects a successful interface improvement. And the reverse saturation current density is also two orders of magnitude smaller than that of control device. The steady‐state PCE is measured at the voltage of maximum power point tracking for 300s in Figure S17 (Supporting Information). The stable PCE is 24.67% for SHA‐treated device at the voltage of 1.02 V without any fluctuation. Even though there is no dramatic decline observed for both devices, the PCE of control device slightly decreases from 23.78% to 23.11%.

The space‐charge‐limited currentmeasurements is used to calculate the trap densities in perovskite thin films by fabricating the electron‐only devices with the configuration of FTO/SnO_2_(SHA–SnO_2_)/perovskite/PC_61_BM/Au. Then, the trap density *N_t_* can be determined by the following equation:

(2) $$N_{t}=\frac{2\varepsilon \varepsilon_{0}V_{TFL}}{qL^{2}}$$

Here, ɛ is the relative permittivity of perovskite layer (ɛ = 32), ɛ_0_ is vacuum permittivity, *q* is the elementary charge, and *L* is the thickness.^[^ ^40^ ^]^ The trap‐filling limited voltage (*V_TFL_*) is obtained from the dark *J*–*V* curves in Figure S18 (Supporting Information). The corresponding *V_TFL_* is 0.69 and 0.81 V for SHA–SnO_2_ and SnO_2_ devices, respectively. Thus, the calculated *N_t_* is 6.78  ×  10^15^ cm^−3^ for SHA–SnO_2_ device and 7.96  ×  10^15^ cm^−3^ for SnO_2_ device. The reduced trap density is ascribed to the higher crystallinity and suppressed non‐radiative recombination of SHA–SnO_2_‐based perovskite films.

Mott–Schottky plot is first performed to study the interfacial carrier dynamics in Figure 5f. The built‐in potential (*V_bi_*) determines the driving force for separating and transferring carrier, which can be obtained by linear fitting the Mott–Schottky plot. The SHA–SnO_2_ device presents a higher voltage of 0.86 V than that of control device (0.73 V). A larger depletion region will form, facilitating the carrier extraction and suppressing the recombination. The transient photocurrent and transient photovoltage are measured to study the carrier transfer and recombination dynamics of devices in Figure 5g,h. The photocurrent decay of SHA–SnO_2_ PSC is faster than SnO_2_ PSC, with the *τ_1_* decreasing from 2.43 to 2.07 µs, reflecting the efficient charge extraction and transfer. Meanwhile, the SHA–SnO_2_ PSC presents a longer recombination lifetime in photovoltage decay (*τ_1_* = 11.8 ms) than the control devices (*τ_1_* = 10.3 ms), suggesting a lower recombination rate and suppressed nonradiative recombination, which matches the enhanced *V*oc. The carrier recombination is suppressed with the help of SHA, which is also proved by electrical impedance spectroscopy (EIS) in Figure 5i’s Nyquist plot, and fitting data is summarized in Table S3 (Supporting Information). The equivalent circuit model is inserted in Figure S19 (Supporting Information), including the series resistance (*R_s_*) and recombination resistance (*R_rec_*). The *R_s_* originates from the high‐frequency region of the plot and is related to the charge transport. While *R_rec_* is extracted from the low‐frequency region and is associated with the recombination rate.^[^ ^41^ ^]^ SHA–SnO_2_ PSC demonstrates a reduced *R_s_* of 14.32 Ω and an increased *R_rec_* of 115.2 Ω, in contrast to the control devices. It is revealed that in the SHA–SnO_2_ PSC, the charge extraction is promoted and the recombination loss is reduced, which is in accordance with the enhanced *V_oc_* and FF above.

To make a comparison, reported works on polymer stabilized SnO_2_ in perovskite solar cells, and other additives are summarized in Figure S20 and Tables S4 and S5 (Supporting Information). Here, SHA devices reached the highest performance either PCE or the detailed photovoltaic parameters, which makes our SHA stands out among all the polymers. Moreover, SHA is a naturally derived, clean and environment‐friendly molecule, supporting its potential for sustainable applications. As summarized in Table S5 (Supporting Information), except for polymers, small molecules, organic salts and acid modifiers are commonly used to treat the SnO_2_. While the working mechanisms of these additives differ, devices incorporating SHA yield highly competitive PCEs. Notably, SHA not only stabilizes the SnO_2_ dispersion, but also regulate the quality of perovskite thin film with oriented growth and reduced the residual strains.

**Figure 6:**
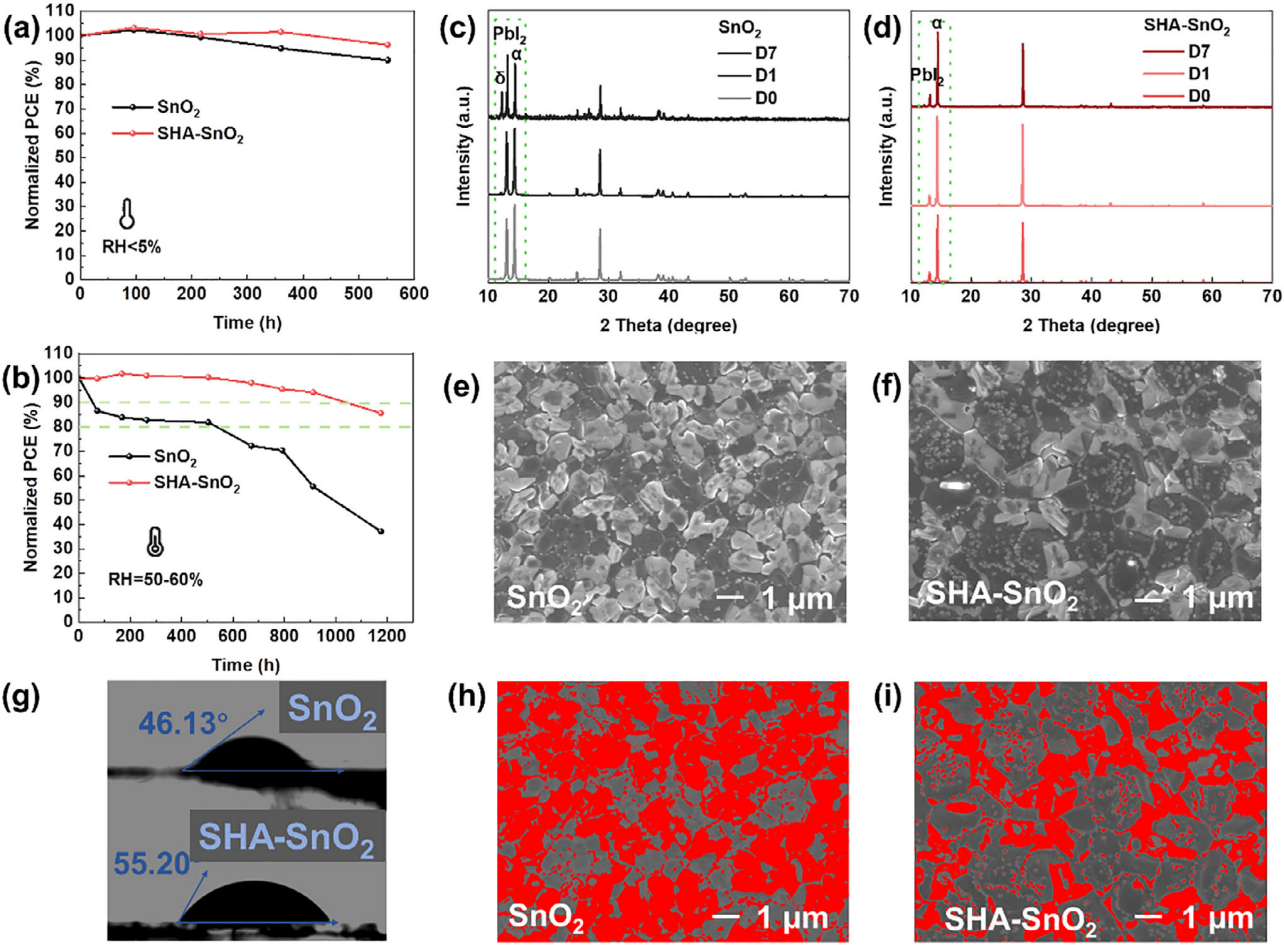
Long‐term stability of the unencapsulated control and target devices. Normalized PCEs of devices stored in a) dDark dry box and b) ambient air without encapsulation; XRD measured at the first day (D0), third day (D3) and seventh day (D7) of perovskite deposited on SnO_2_ c) and SHA–SnO_2_ d) films stored in ambient air with humidity over 50% RH; Top‐view SEM images of perovskite films on SnO_2_ e) and SHA–SnO_2_ f) stored in ambient air for 3 days; g) contact angle of perovskite films; h,i) highlighted SEM images via Image J software, where the red part is degraded area.

Long‐term stability of devices, the PSCs with or without SHA are stored in a dark dry box (humidity: ≈1–5%, temperature: ≈20–30 °C). Both of them can retain the performance at the relevantly optimal level in **Figure** **6**a, while the PCE of control device slightly decreases to its 90% after 500 h storage. The PCE decay at ambient environment (humidity: ≈50–60%, temperature: ≈20–30 °C) without encapsulation is exhibited in Figure 6b. Once placed the devices at the wet environment without protection, the SHA–SnO_2_ device shows superior advancement in the PCE tracking, which still maintain 90% of its original efficiency after 1000 h. The operational stability under maximum power point is measured in ambient air with continuous illumination (AM 1.5G, T ≈ 55 °C) without encapsulation. The control device drops to 40 % of its original PCE after 2 days (Figure S21, Supporting Information) at such harsh condition. While the devices based on SHA still retain above 60% over one week. To further investigate the degradation mechanism and process, XRD in Figure 6c,d is used to track the crystallinity changes of perovskite thin films deposited on top of SnO_2_ and SHA–SnO_2_ when put in moisture atmosphere for one week. The control film has decomposed over 1 day when exposed to the humidity, leading to the increased intensity of PbI_2_ peaks. Even worse, the δ‐phase has appeared after 7days while the SHA‐modified one still retain the black α‐phase. The degradation always starts from the imperfection of perovskite like the pin‐holes and grain boundaries. The existence of void at the interfaces and in the bulk of perovskite in Figure 3c,d accelerates the degradation of control film as well. It is indicated that the SHA in SnO_2_ hinders the phase transition under moisture environment, which acts as a bridge to anchor the perovskite tightly with the ETL, leading to reduced voids and excellent crystallinity. The improvement on tolerance for moisture may also benefit from the more hydrophobic perovskite in Figure 6g, which prevents the water penetrating into the perovskite. Figure 6e,f exhibits the effects on morphological changes of perovskite thin films from SEM images after 3 days storage. In comparison with the fresh thin film in Figure 3a,b, the white PbI_2_ domain appears at the surface, which is highlighted in Figure 6h,i. And the histograms of the area ratios of white domains calculated by ImageJ software in Figure S22 (Supporting Information). More than 60% of control thin film turns white when the corresponding area of SHA–SnO_2_‐based perovskite film is less than 40%.

## 2 Conclusion

In sum, a compact and homogeneous SnO_2_ ETL is prepared by applying SHA into SnO_2_ dispersion. SHA maintain the long‐term stability of SnO_2_ nanoparticles and prevent the agglomeration by electrostatic force and steric effects. It also eliminate the hydroxyl groups and oxygen vacancies at the interface, promoting a well‐aligned energy level and reducing carrier accumulation. Benefiting from the modification of buried contact interface, the obtained perovskites grains grow vertically to the substrate with reduced voids. As a result, the PSC device fabricated on SHA–SnO_2_ demonstrates a champion PCE as high as 25.11%, owing to enhanced *V_oc_* and FF. More importantly, the unencapsulated devices still maintain 90% of its original PCE after stored at the ambient air with humidity over 50% for 1000 h and retain 60% PCE after 200 h under continuous light soaking for MPPT tracking with temperature over 55 °C. It provides an effective strategy for in situ stabilizing the SnO_2_ nanoparticles in future large‐scale commercialization.

## Conflict of Interest

The authors declare no conflict of interest.

## Supporting information



Supporting Information

## Data Availability

The data that support the findings of this study are available from the corresponding author upon reasonable request.
