# Supplementary material for: Multifunctional Polymer Matrix at the Buried Interface Boosting Stability and Efficiency in Perovskite Solar Cells
Source: Small. 2025 Sep 27;21(46):e07718. doi: 10.1002/smll.202507718 (PMC12632439; doi:10.1002/smll.202507718)
Supplement: Supplementary file 1 — Supporting Information [file SMLL-21-e07718-s001.docx]

**Multifunctional Polymer Matrix at the Buried Interface Boosting Stability and Efficiency in Perovskite Solar Cells**

Huiming Luo, Zhijie Gao, Himal Muwanwella, Galyam Sanfo, Ying Lu, Shurui Yang, Yinheng Ren, Shanyue Hou, Xiang Liu, Preetam Kumar Sharma, Robert Palgrave, Muhammad Tariq Sajjad*, Peng Huang*, Mojtaba Abdi-Jalebi*

Huiming Luo, Galyam Sanfo, Ying Lu, Shurui Yang, Preetam Kumar Sharma, Mojtaba Abdi-Jalebi.

Institute for Materials Discovery, University College London, Malet Place, London, WC1E 7JE, UK.

E-mail: [m.jalebi@ucl.ac.uk](mailto:m.jalebi@ucl.ac.uk)

Zhijie Gao, Peng Huang.

Key Laboratory of Advanced Technologies of Materials, Ministry of Education, School of Materials Science and Engineering, Southwest Jiaotong University, Chengdu, 610031, PR China.

Research Institute of Frontier Science, Southwest Jiaotong University, Chengdu 610031, PR China.

E-mail: [phuang@swjtu.edu.cn](mailto:phuang@swjtu.edu.cn)

Himal Muwanwella, Muhammad Tariq Sajjad

School of Engineering and and Design, London South Bank University, 103 Borough Road, London, SE1 0AA UK.

E-mail: [sajjadt@lsbu.ac.uk](mailto:sajjadt@lsbu.ac.uk)

Robert Palgrave

Department of Chemistry, University College London, London WC1H 0AJ, U.K.

Yinheng Ren, Shanyue Hou, Xiang Liu

School of Materials Science and Engineering, Beihang University, Beijing 100084, P. R. China.

^#^Huiming Luo and Zhijie Gao contributed equally to this work.

**1 Experimental section**

**Materials:**

MACl (99.5%), FAI (99.5%), PEAI (99.5%) are purchased from Greatcell Solar Ltd. PbI_2_ (99.99%) is purchased from Tokyo Chemical Industry UK Ltd.. CsI (99.99%) is purchased from Alfa Aesar. Isopropanol (IPA, 99.5%), N, N-dimethylformamide (DMF, anhydrous, 99.8%), dimethyl sulfoxide (DMSO, anhydrous, 99.8%), chlorobenzene (CB, anhydrous, 99.8%). The SnO_2_ colloid precursor (tin (IV) oxide, 15% in H_2_O colloidal dispersion) was acquired from Alfa Aesar. Sodium hyaluronate, 95% Fisher Scientific Ltd FTO glasses (15 Ω sq^-1^) were purchased from Advanced Election Technology Co. Ltd. All of these commercially available materials are used as received without further purification.

**Device fabrication:**

FTO substrates are washed with detergent, deionized water, acetone, ethanol, and isopropanol for 10 min in an ultrasonic bath twice, followed by 10 min UV ozone treatment before use. The SnO_2_ colloid solution was diluted with water in a ratio of 1: 6 in volume, and spun-coated on FTO in ambient air with the rate of 3000 rpm for 30s, and then annealed at 150 °C for 30 min. The perovskite layer was prepared by a two-step method. For the perovskite precursor, 1.3 M PbI_2_ was dissolved in a mixture of DMF and DMSO (19:1) with addition of 5% CsI. While 60 mg/mL FAI was mixed with 20% MACl in IPA. After cooling down, the perovskite layer was deposited through spin-coating the perovskite precursor onto SnO_2_/FTO substrates at 2000 rpm for 30 s, following by an annealing process at 70 °C for 1 min. After that, the FAI was poured on substrate with the same program. The substrate was transferred to a hot plate and annealed at 150 °C for 15 min. Then, 2 mg/mL PEAI in IPA was spun on it at a speed of 5000 rpm for 30s. For the preparation of hole transport layer, 2,2′,7,7′-tetrakis(N, N-di-pmethoxyphenylamine)-9, 9′-spirobifluorene (Spiro-OMeTAD) solution consisted of 72.3 mg of spiro-OMeTAD, 29 μL of 4-tert-butyl pyridine (tBP), 18 μL of lithium bis(trifluoromethanesulfonyl)imide (Li-TFSI) solution (520 mg of Li-TSFI in 1 mL acetonitrile) in 1 mL of chlorobenzene. It was spin-coated on the perovskite layer after it totally cooled down to room temperature at 3000 rpm for 30 s. Finally, Au electrode with a thickness of 80 nm was deposited by using a thermal evaporator at the rate of 0.1 Å∙s^-1^.

**Characterization:**

UV-vis absorption spectrum of the material was recorded on PerkinElmer Lambda 750S when the test environment was room temperature, the wavelength range was 350-900 nm and the slit width was 1 nm. DLS was conducted on the light scattering from Malvern Panalytical Zetasizer Lab.

The top-view and cross-sectional SEM images of the SnO_2_ samples were characterized by Zeiss SUPRA 55-VP and the perovskite thin films were measured by using a field emission scanning electron microscope (JEOL 7600F) in high-vacuum and high-resolution mode. AFM was carried by Nanosurf CoreAFM with Isostage 300. Transmission Electron Microscopy (TEM) was tested by A Jeol 2100 200 kV fitted with a LaB6 filament giving a point resolution of 0.13 nm.

*J-V* characteristics of photovoltaic cells were taken using a Keithley 2400 source measurement unit under a simulated AM 1.5G spectrum with a solar simulator from Newport Corporation Oriel® Sol1A; the light intensity was calibrated by standard single-crystal silicon solar cells. The corresponding IPCE spectra were measured on a Quantum Efficiency Measurement System at room temperature in ambient conditions, by using a Xe-lamp light source (Enlitech QE-R), and calibrated by standard silicon cells. X-ray diffraction (XRD) and Grazing incidence X-ray diffraction (GIXRD) were measured on AERIS Panalytical Research Edition. Steady Photoluminescence (PL) was measured by Ocean View (Ocean insight) and time-resolved photoluminescence (TRPL) was measured by FLS1000 Photoluminescence Spectrometer (Edinburgh Instruments) with an excitation wavelength of 405 nm.The X-ray photoelectron spectroscopy (XPS) is measured by a Kratos Analytical AXIS Ultra DLD spectrometer (Harwell XPS facility) equipped with a monochromatic Al Kα (1486.6 eV) source, operating under UHV conditions. Detailed spectral analysis was performed by using CasaXPS, with binding energy calibration referenced to the C 1s peak at 284.8 eV. Ultraviolet photoelectron spectroscopy (UPS) is measured by the same equipment. A He discharge lamp to provide ultraviolet photons at an energy of 21.22 eV. Transient Photocurrent (TPC), Transient photovoltage (TPV) and Electrochemical Impedance Spectroscopy (EIS) were measured by Paios measurement platform (Fluxim AG).

**DFT calculation**

All calculations use the Cambridge Serial Total Energy Package (CASTEP) code in Materials Studio. The generalized gradient approximation (GGA) of Perdew-Burke-Ernzerhof (PBE) functional was used to handle the exchange correlation energy between electrons, which has been proven to have quite good accuracy and efficiency in the process of geometry optimization. To simulate the adsorption process between molecules and crystal surfaces, we constructed 4×2 and 4×3 supercells for FAPbI_3_ (001) and SnO_2_ (001), respectively. The 20Å vacuum slab was added in the z-axis direction to isolate intermolecular interactions caused by periodic principles. The van der Waals interaction was taken into account through the use of the DFT-D3 method. Due to the large number of atoms in the model, in order to maximize the calculation speed while ensuring accuracy, the plane-wave cut-off energy was set to 400 eV, and 1×2×1 Monkhorst-Pack K-point sampling was used in the Brillouin zone. The process of geometry optimization is considered completed when all ions’ energy were less than 10^-4^eV, and residual force is less than 0.03eV. The adsorbing energy can be calculated using the following formula：

$$E\text{a}\text{d}\text{s}=E\text{surface}+\text{molecule}-E\text{molecule}-E\text{surface}$$

where *E_molecule_*, *E_surface_*, and *E_surface+molecule_* are the energies of the adsorbing molecule, FAPbI_3_/SnO_2_ surface, and final system, respectively.


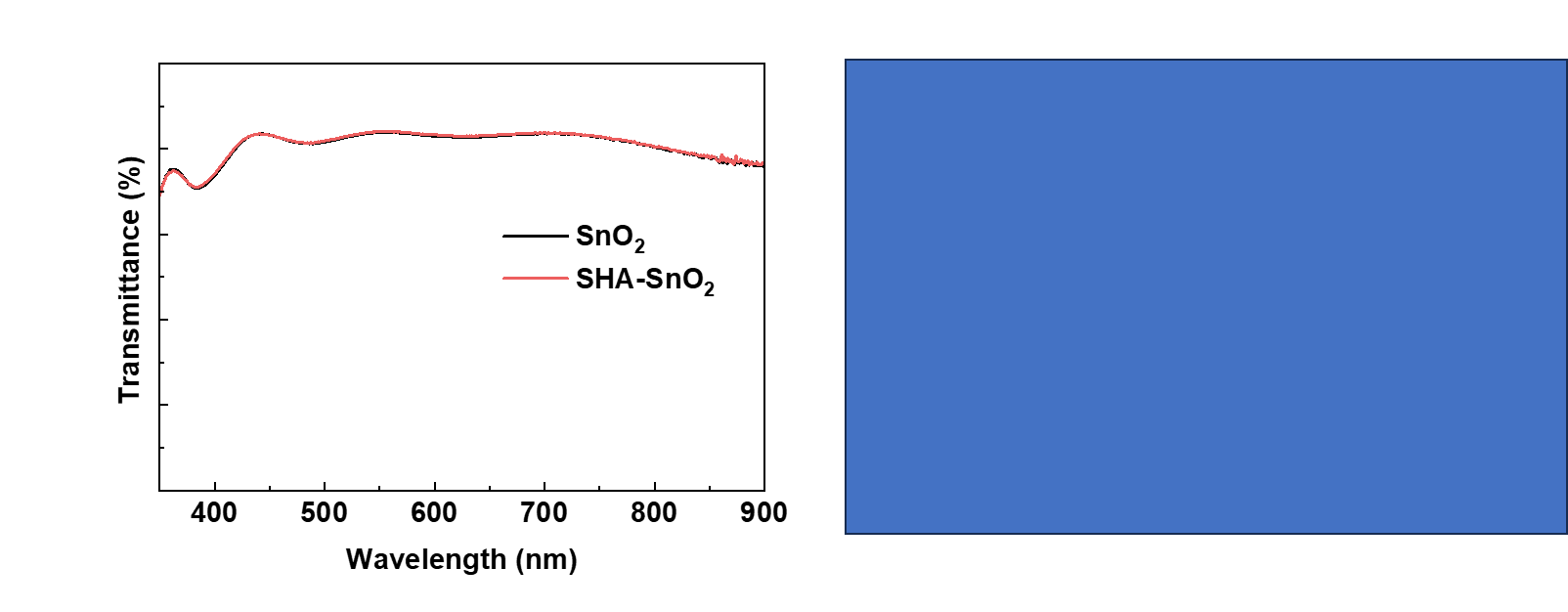


**Figure S1**. Transmittance spectrum of SnO_2_, and SHA-SnO_2_ thin films on FTO in the wavelength range of 350-900 nm using a UV–vis spectrophotometer.


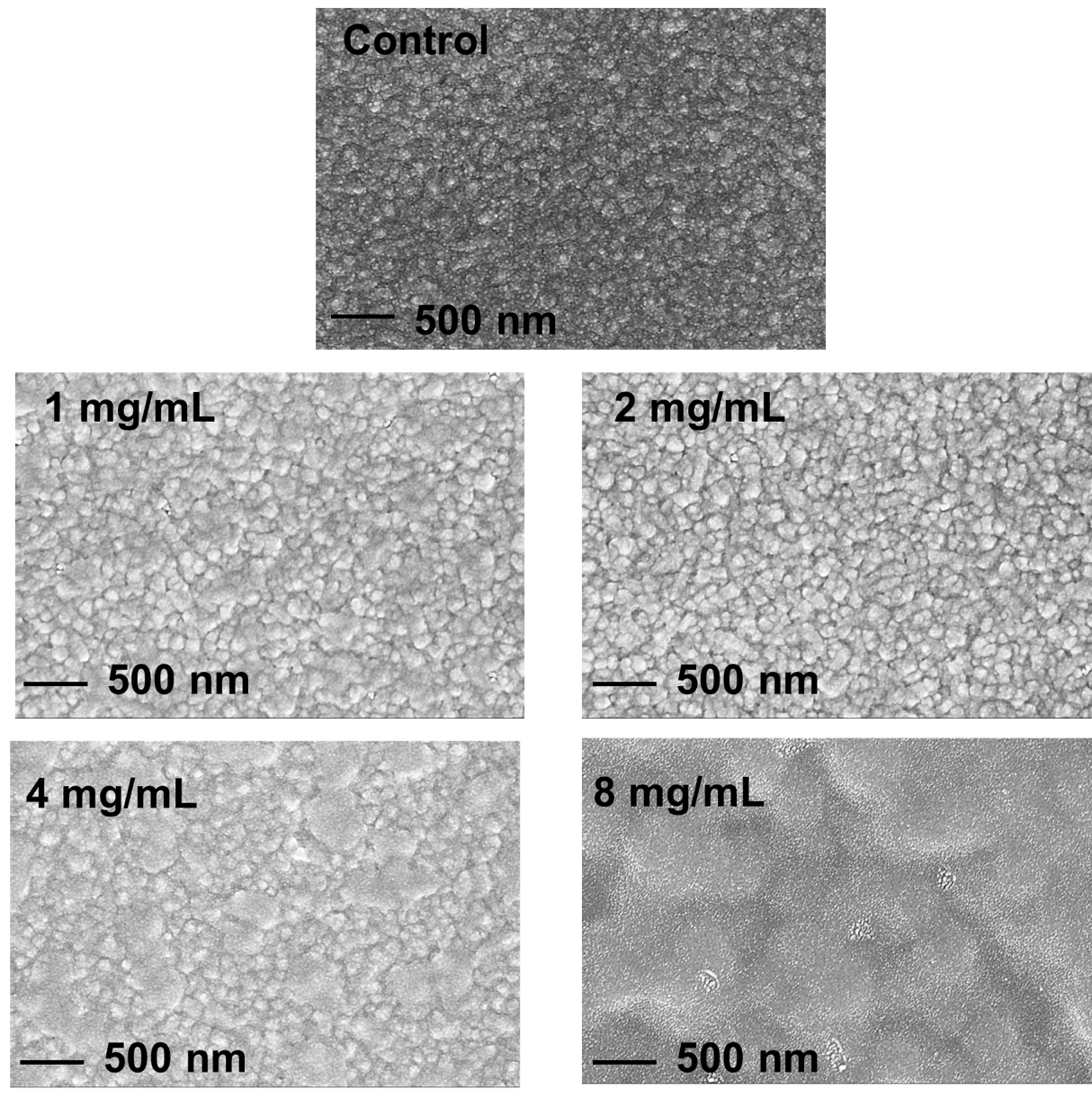


**Figure S2**. Top-view SEM images of SnO_2_ thin films with different concentrations (0-8 mg mL^-1^) of SHA. The comparison demonstrates the effect of SHA incorporation on the surface morphology, where at higher concentrations (>4 mg mL^-1^), noticeable particle agglomeration and surface clustering are observed.


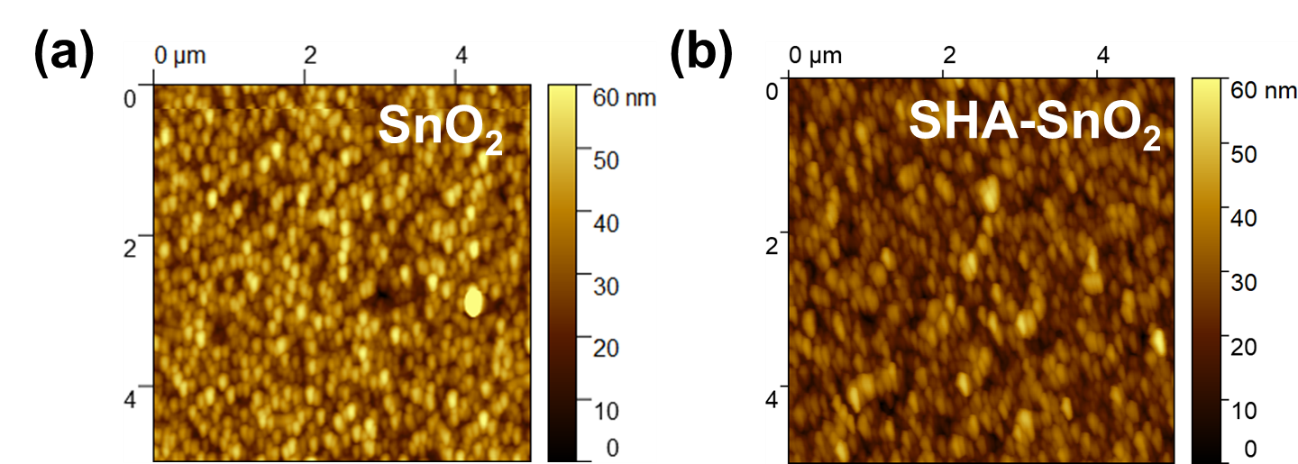


**Figure S3**. AFM images of SnO_2_ and SHA-SnO_2_ thin films.


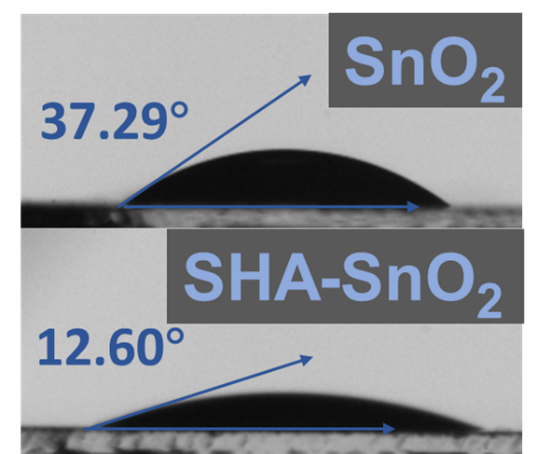


**Figure S4**. Water contact angle of SnO_2_ thin film with and without SHA, performed at room temperature using a sessile drop method.


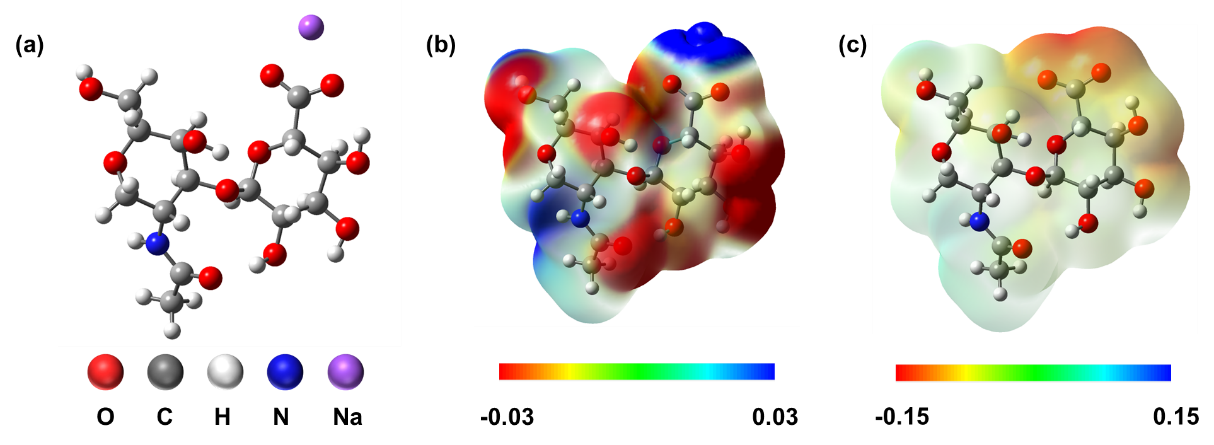


**Figure S5**. (a) Chemical structure of SHA; Electrostatic potentials of SHA with (b) or without (c) Na^+^.


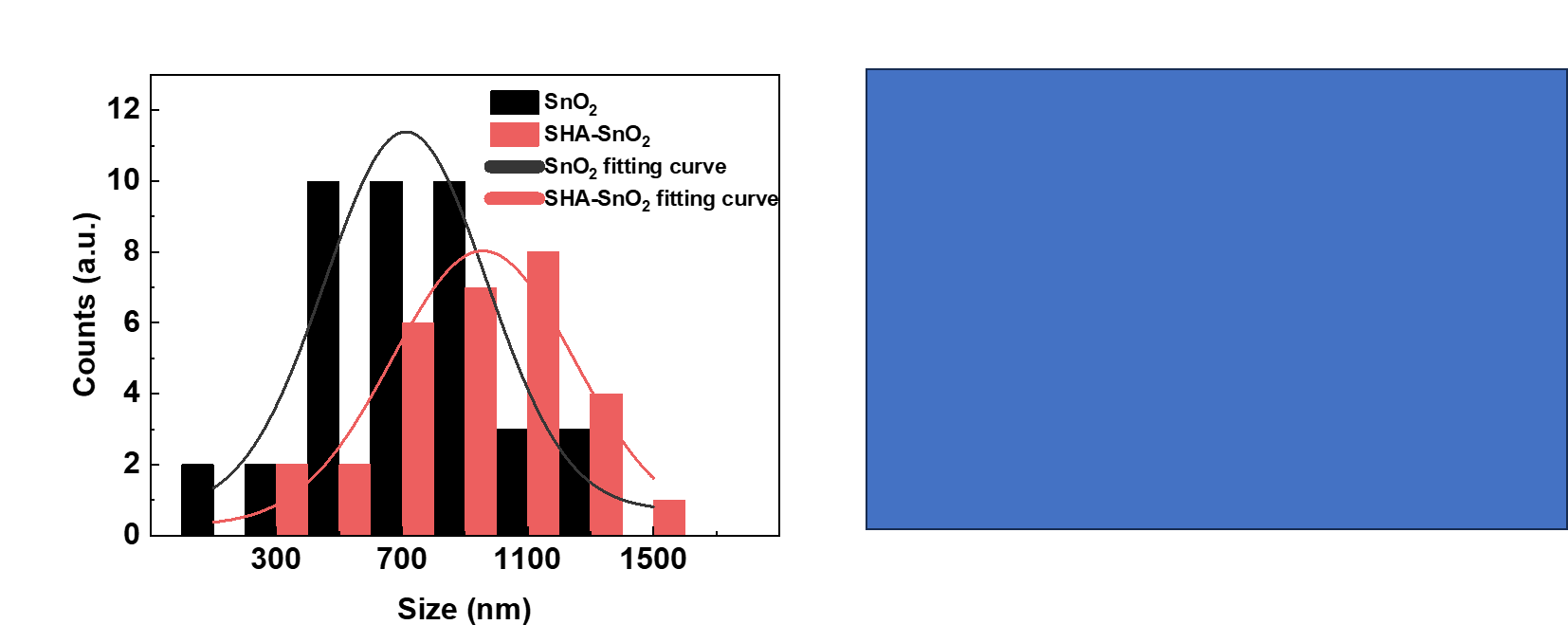


**Figure S6**. Grain size distributions from top-view SEM images of perovskite films in Figure 3(a-b).


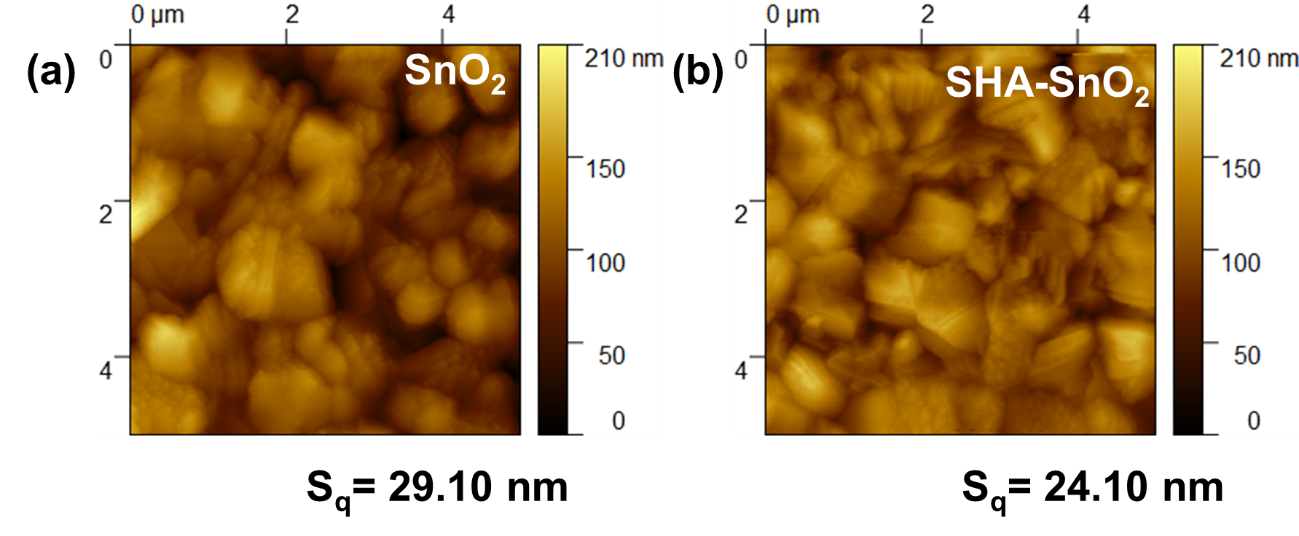


**Figure S7**. AFM images of perovskite thin films deposited on the SnO_2_ and SHA-SnO_2_. The measurements were performed in tapping mode with a scan area of 5 × 5 μm². The images reveal that SHA modification of the SnO₂ layer results in smoother perovskite surfaces with reduced roughness and fewer pinholes.


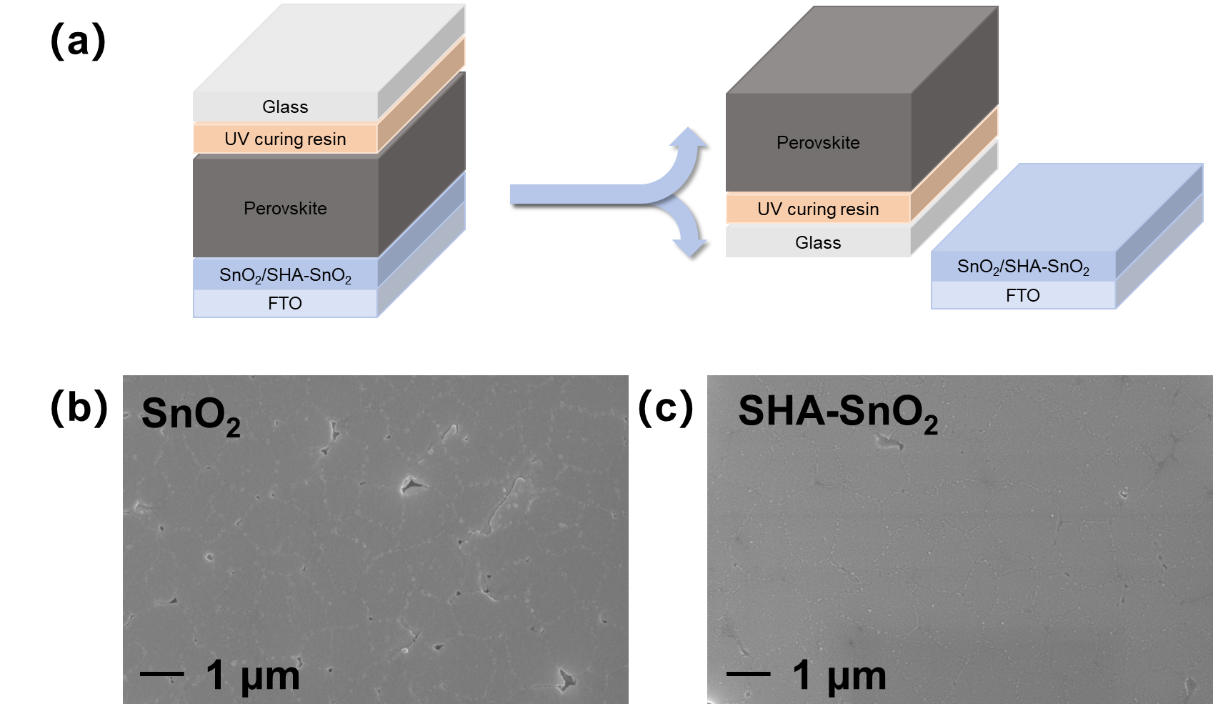


**Figure S8.** Illustration of peel-off method: The UV-curing resin is deposited onto the perovskite thin film, covered with a glass slide, and subsequently solidified under UV illumination.


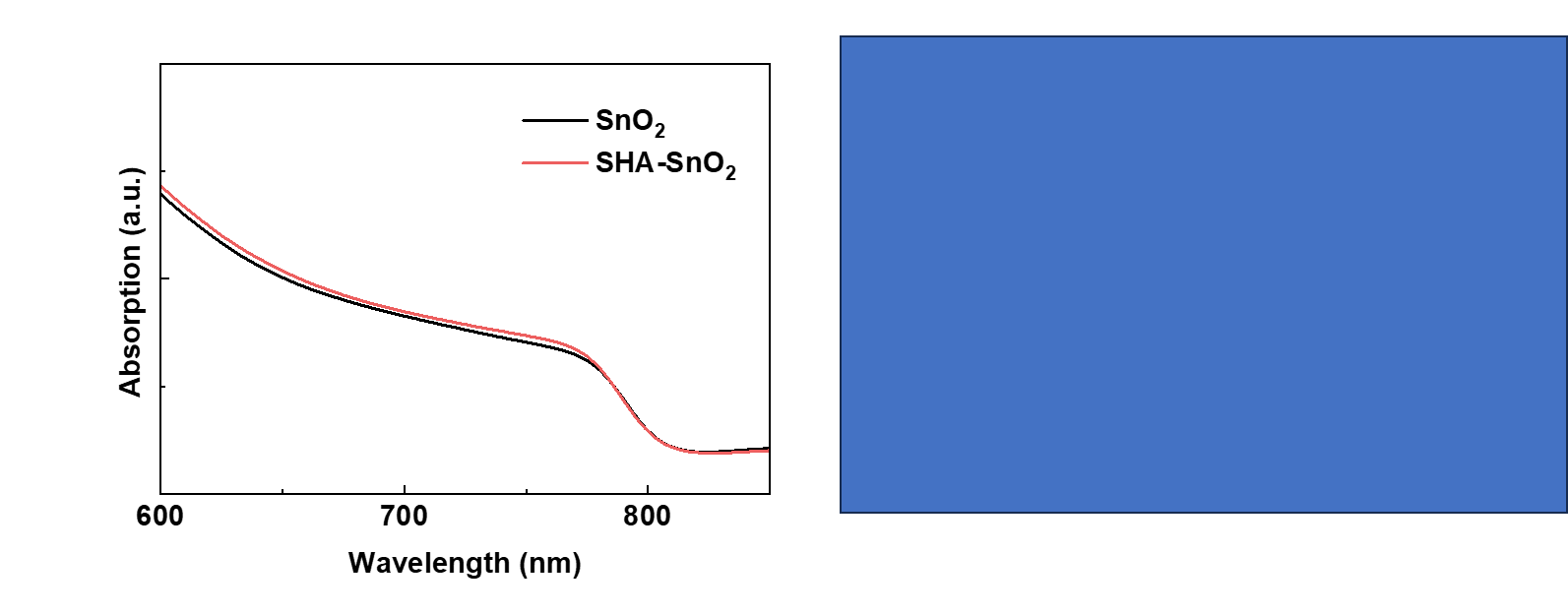


**Figure S9**. UV-vis spectra of perovskite films deposited on the SnO_2_ and SHA-SnO_2_.


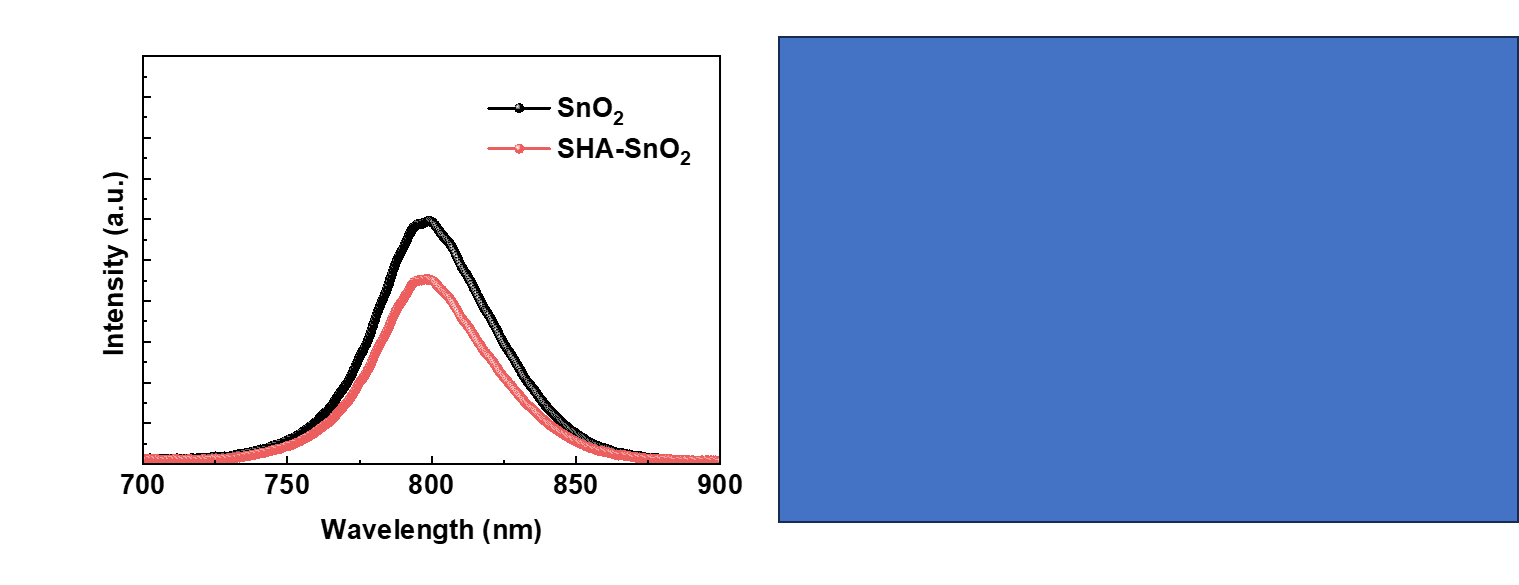


**Figure S10**. PL spectra of perovskite films deposited on the SnO_2_ and SHA-SnO_2_ with an excitation wavelength of 405 nm. Films on SHA-SnO₂ exhibit reduced PL intensity compared with those on pristine SnO₂, indicating more efficient charge extraction at the interface due to SHA modification.


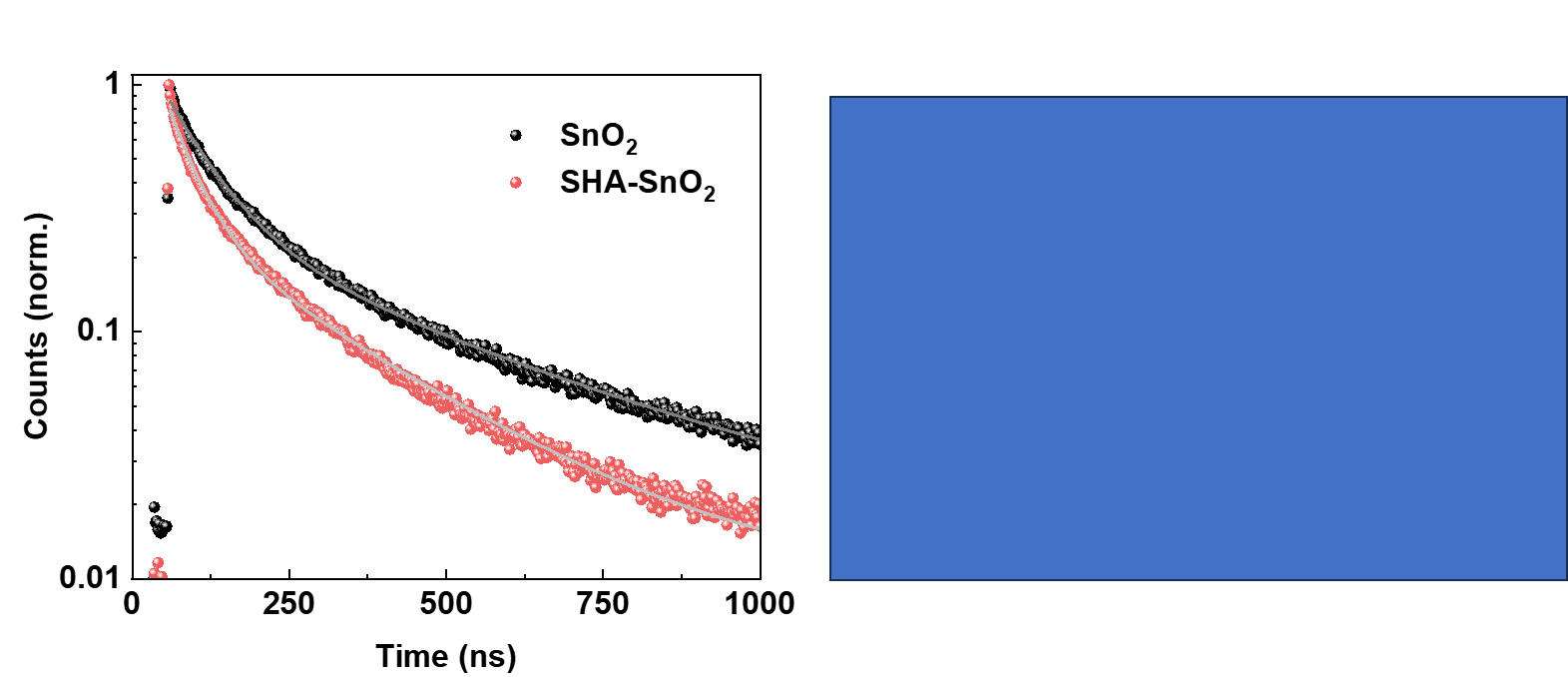


**Figure S11**. TRPL spectra of perovskite films deposited on the SnO_2_ and SHA-SnO_2_ with an excitation wavelength of 405 nm.


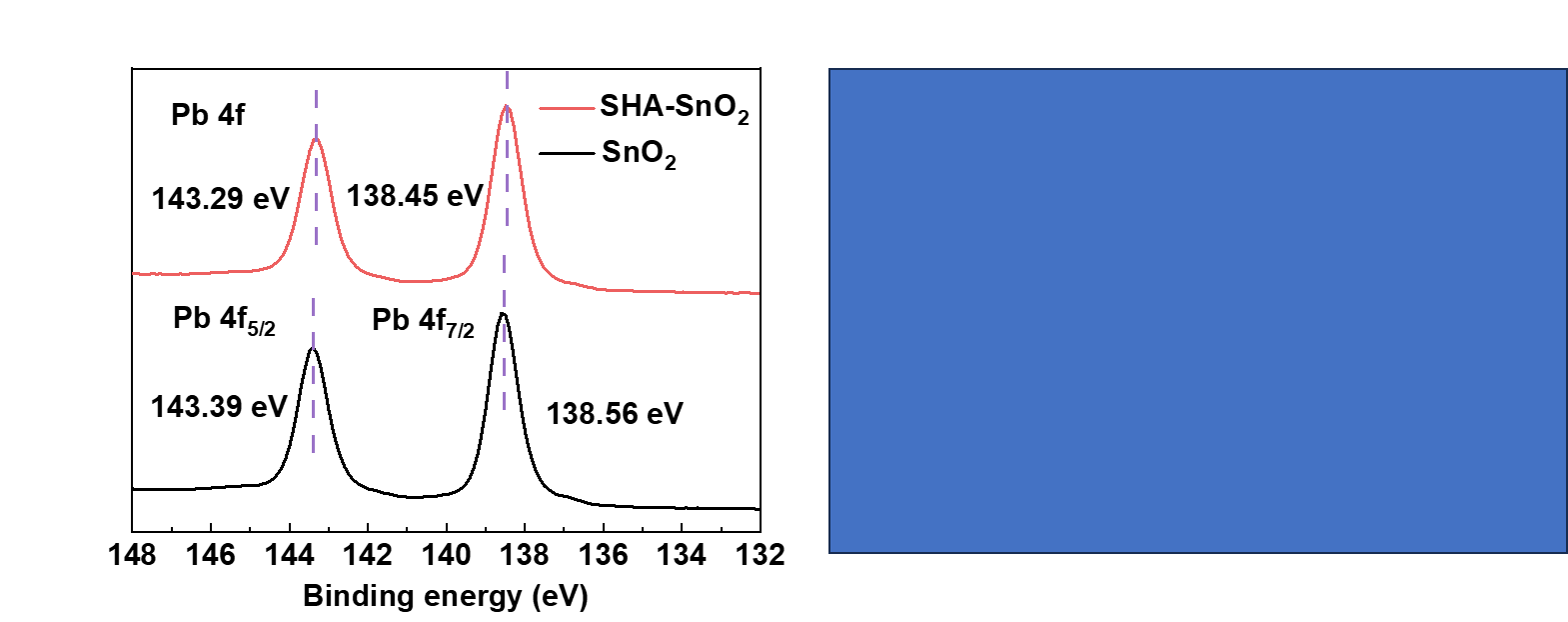


**Figure S12.** XPS spectra of Pb 4f peaks in perovskite films on SnO_2_ and SHA-SnO_2_.


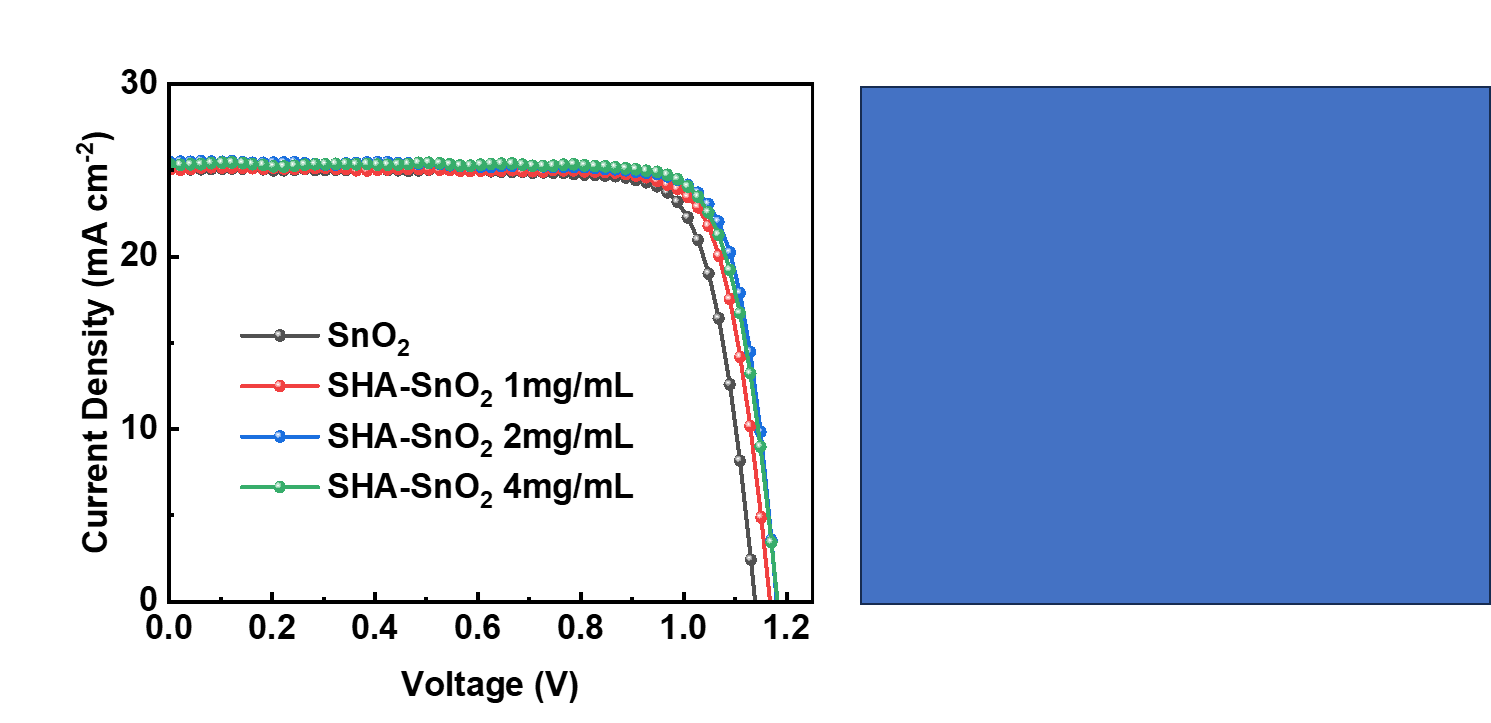


**Figure S13**. *J-V* curves of devices fabricated with different SHA additive concentrations in SnO_2_ solutions from the same batch.


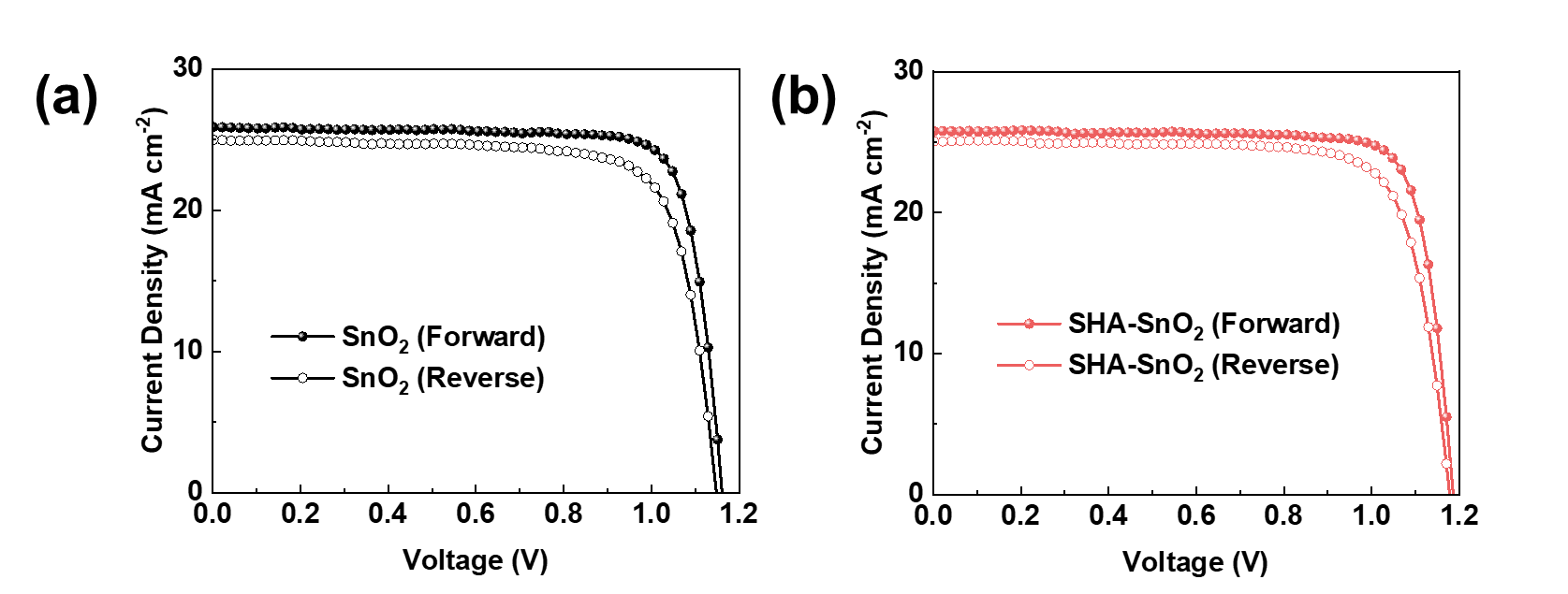


**Figure S14**. *J-V* curve of devices based on SnO_2_ (a) and SHA-SnO_2_ (b) scanned at forward and reverse directions under 1 sun.


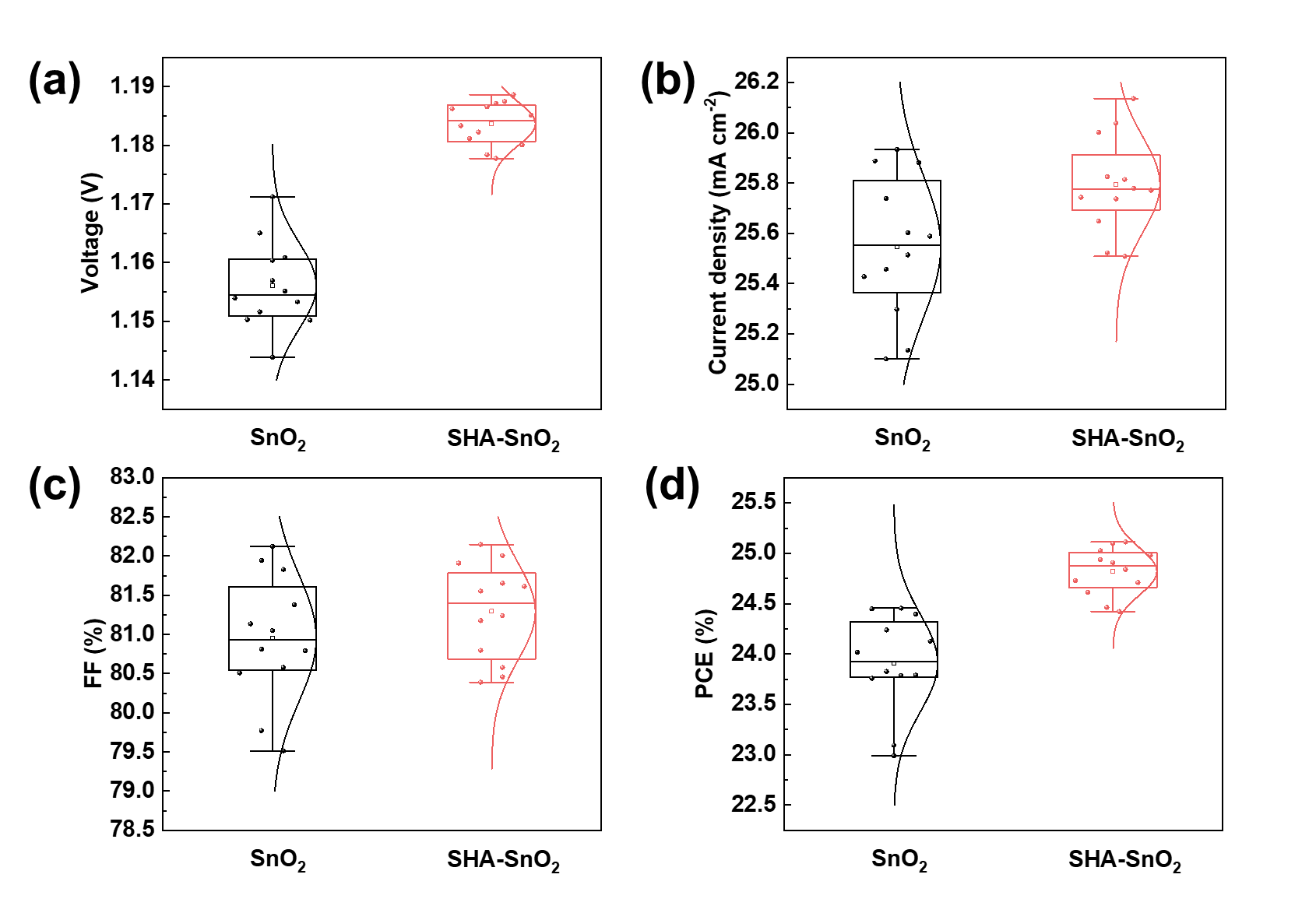


**Figure S15**. Statistics of photovoltaic parameters *V_oc_* (a), *J_sc_* (b), *FF* (c), and *PCE* (d) with 12 pixels.


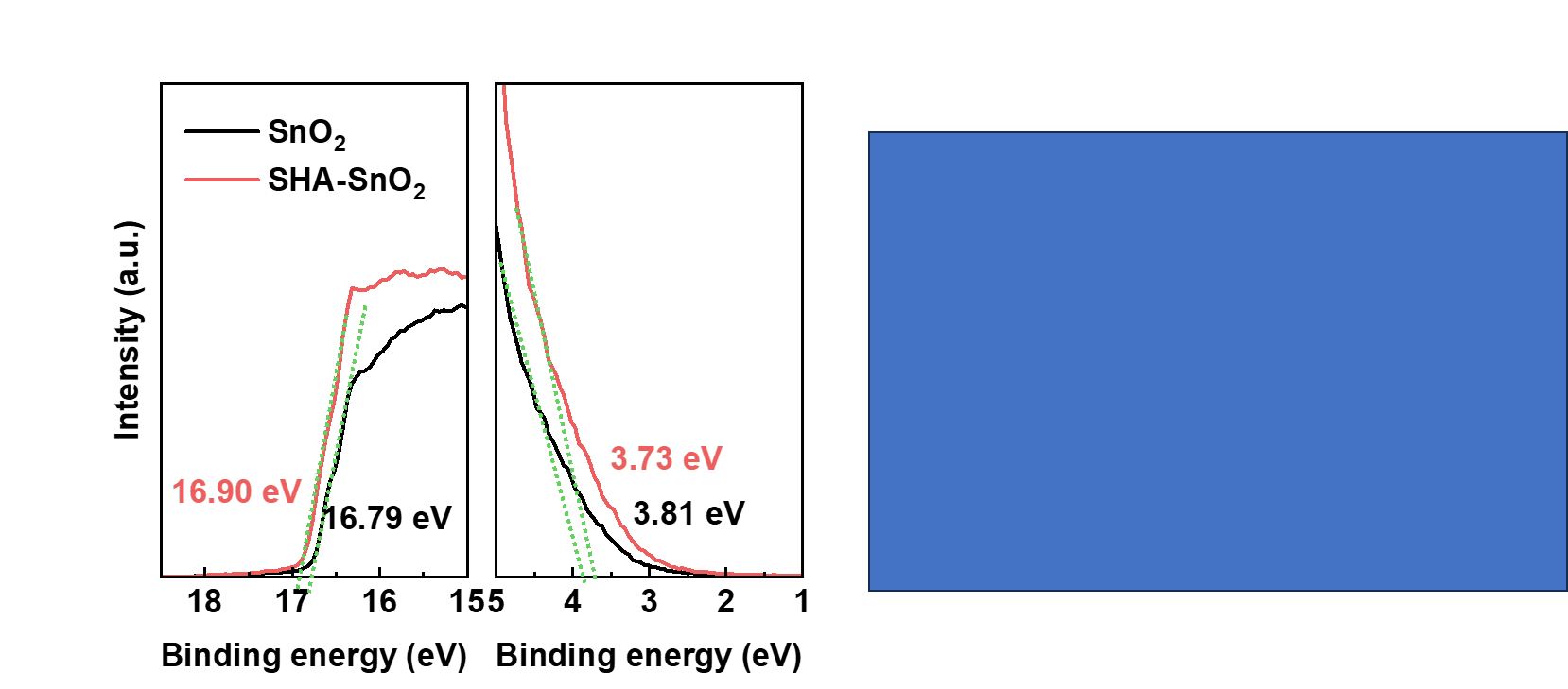


**Figure S16**. UPS spectra of SnO_2_ and SHA-SnO_2_ thin films with onset and cutoff labelled to determine the work function and valence band edge positions.


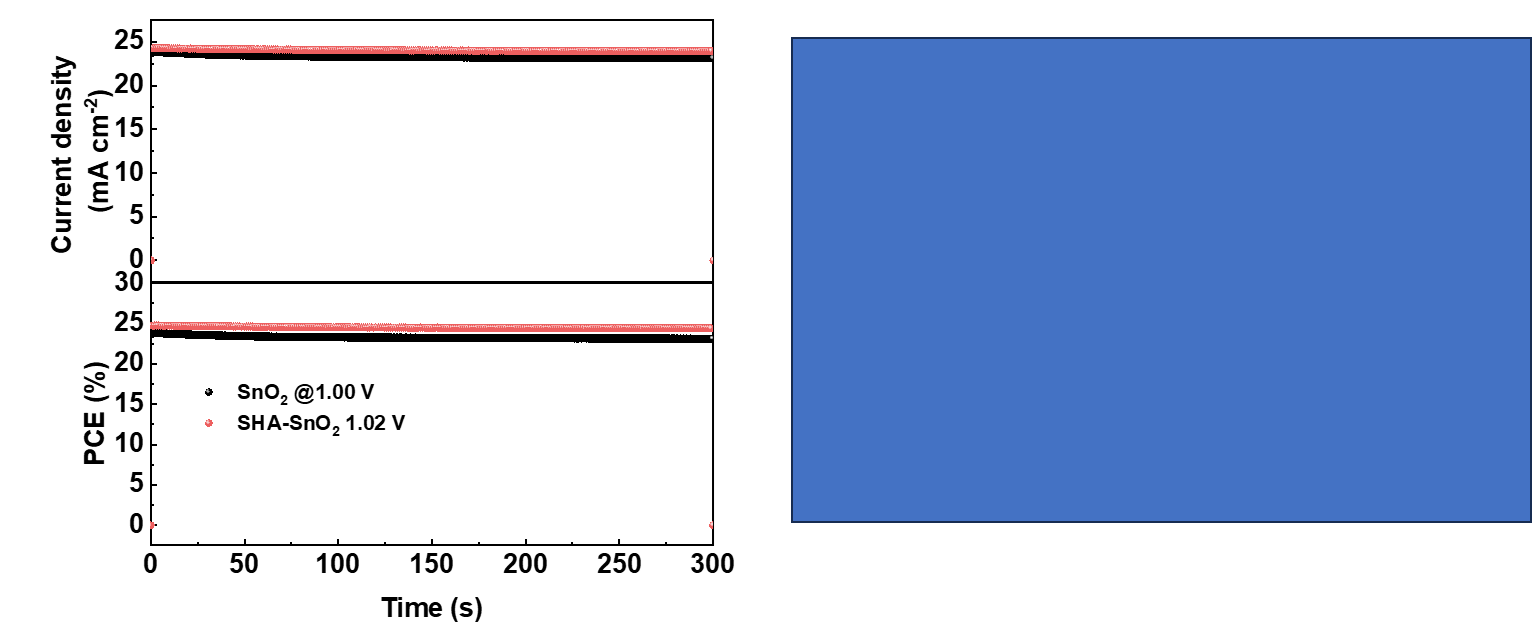


**Figure S17**. Maximum power point tracking of perovskite solar cells with SnO_2_ and SHA-SnO_2_ electron transport materials.


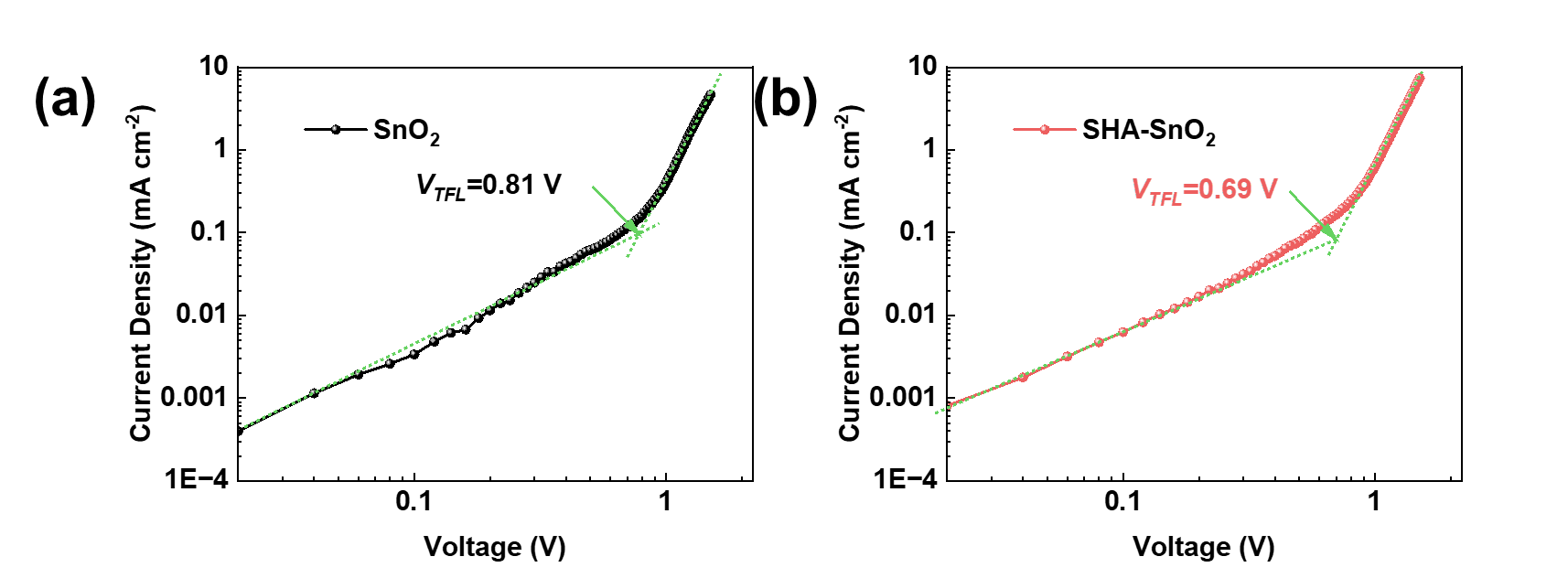


**Figure S18**. Dark *J–V* measure curves of the electron-only devices with the structure of FTO/SnO_2_(SHA-SnO_2_)/Perovskite/PCBM/Au.


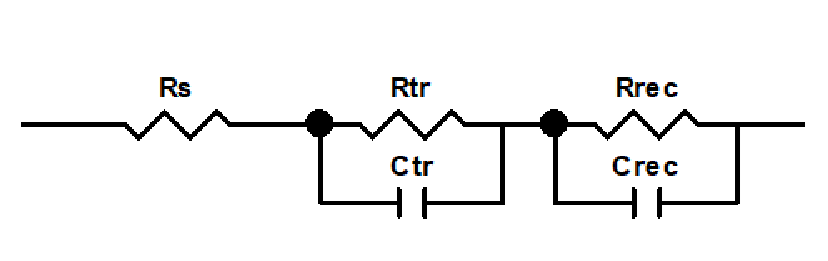


**Figure S19**. Equivalent circuit for fitting Nyquist plot, consisting of series resistance (*R_s_*), charge transfer resistance (*R_tr_*), charge transfer capacitance (*C_tr_*), recombination capacitance (*C_rec_*) and recombination resistance (*R_rec_*) and the relevant parameters are listed in Table S3.


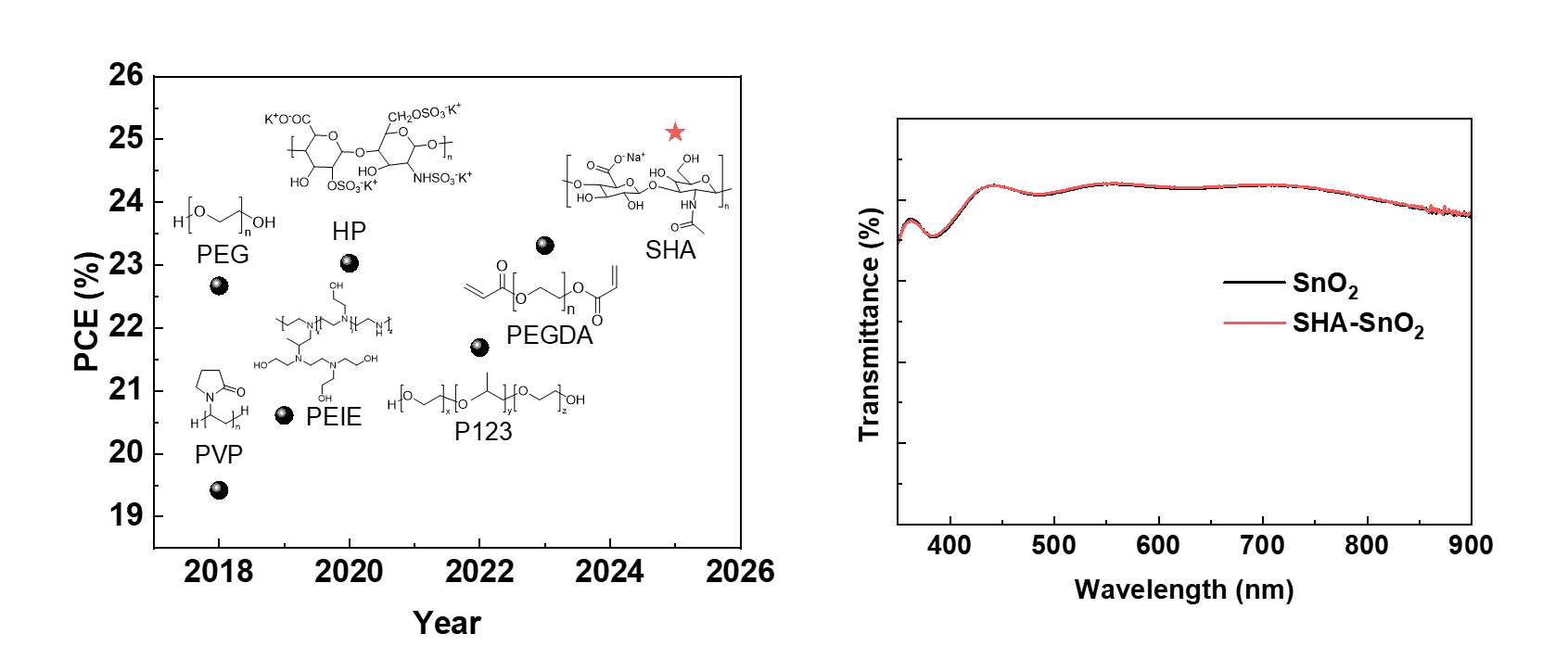


**Figure S20.** Summary of PCEs of polymers stabilized SnO_2_ in perovskite solar cells.


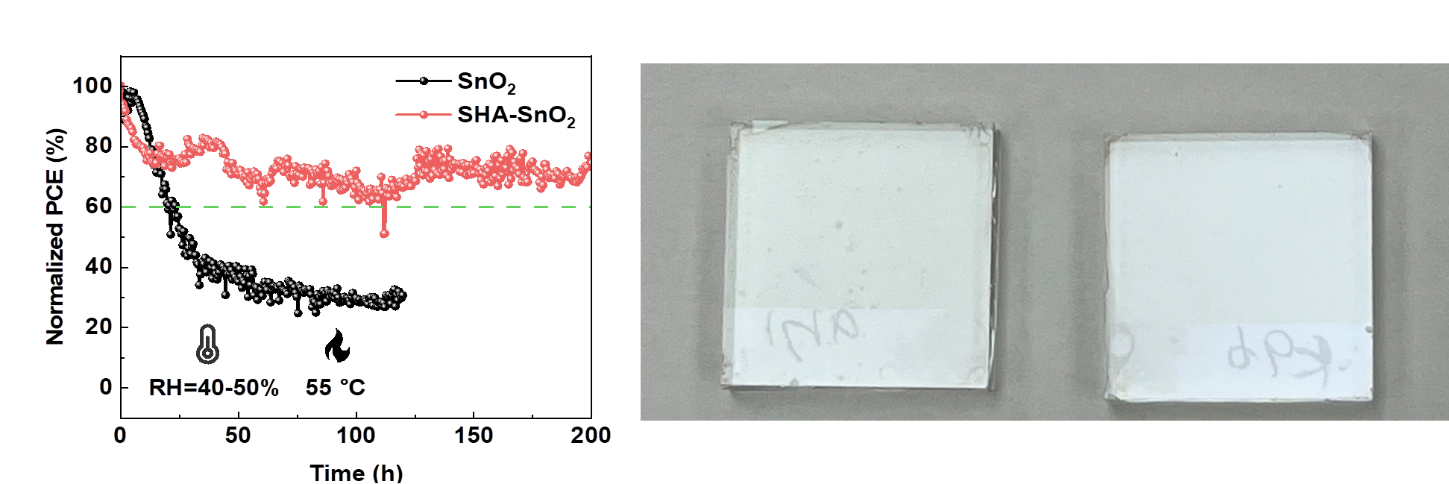


**Figure S21.** Maximum power point tracking (MPPT) of perovskite solar cell unencapsulated devices measured at 55 °C under continuous one-sun illumination in ambient air (AM 1.5G, 100 mW cm⁻²).


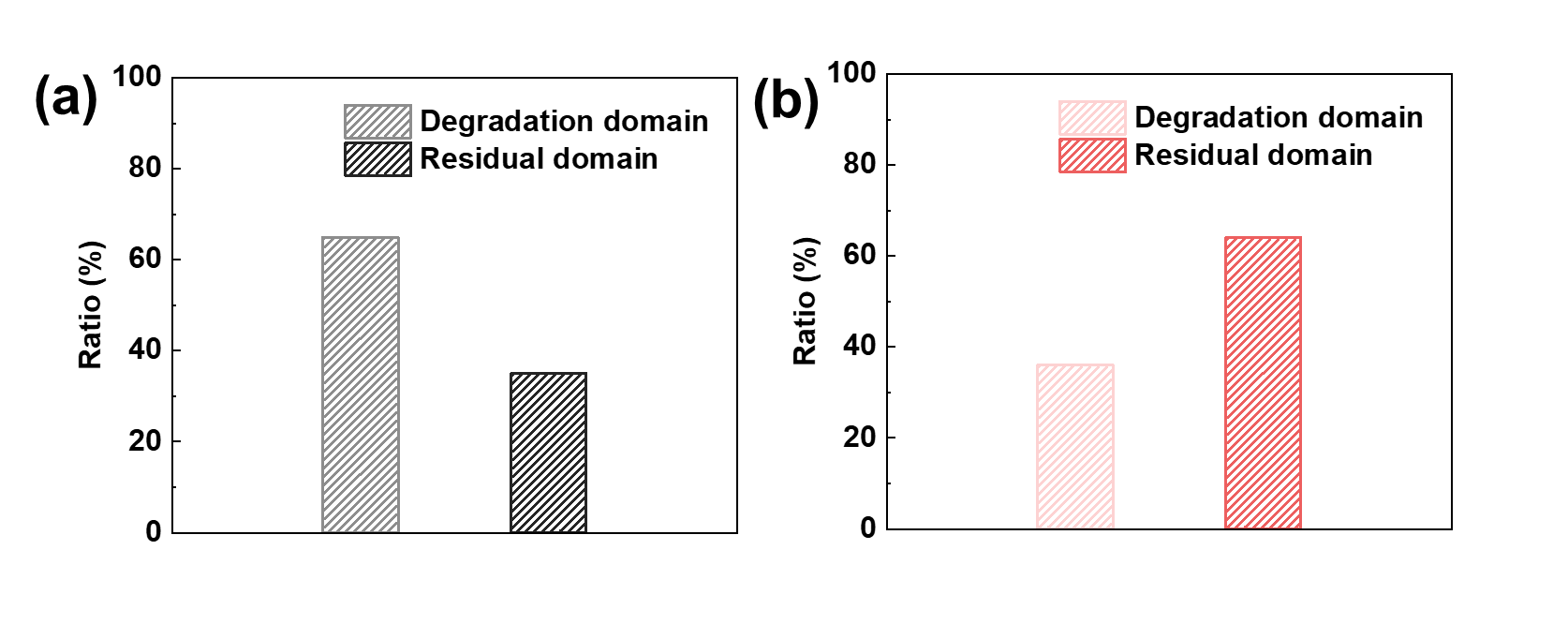


**Figure S22**. Histograms of degradation area calculation of SnO_2_ (a) and SHA-SnO_2_ (b) based perovskite thin films.

**Table S1**. Photovoltaic parameters of solar cells based on different SHA concentrations.

| **SHA Concentration** | ***V_oc_* (V)** | ***J_sc_* (mA/cm^2^)** | ***FF* (%)** | ***PCE* (%)** |
| --- | --- | --- | --- | --- |
| 0 mg/ml | 1.1 | 25.14 | 80.23 | 22.97 |
| 1 mg/ml | 1.17 | 25.06 | 80.80 | 23.65 |
| 2 mg/ml | 1.18 | 25.51 | 80.89 | 24.37 |
| 4 mg/ml | 1.18 | 25.36 | 80.91 | 24.26 |

**Table S2**. Photovoltaic parameters of champion devices at forward and reverse scanning directions and average parameters based on 12 devices.

| **Samples** |  | ***V_oc_* (V)** | ***J_sc_* (mA cm^-2^)** | ***FF* (%)** | ***PCE* (%)** |
| --- | --- | --- | --- | --- | --- |
| SnO_2_ | Forward | 1.15 | 24.97 | 76.77 | 22.01 |
|  | Reverse | 1.16 | 25.88 | 81.38 | 24.45 |
|  | Average | 1.16 | 25.55 | 80.95 | 23.91 |
| SHA-SnO_2_ | Forward | 1.18 | 25.06 | 77.86 | 22.98 |
|  | Reverse | 1.19 | 25.77 | 82.15 | 25.11 |
|  | Average | 1.18 | 25.84 | 81.22 | 24.83 |

**Table S3**. EIS parameters, fitted with ZView2 software.

| **Samples** | **R_s_ (Ω)** | **R_tr_ (Ω)** | **C_tr_ (F)** | **R_rec_ (Ω)** | **C_rec_ (F)** |
| --- | --- | --- | --- | --- | --- |
| SnO_2_ | 15.97 | 23.49 | 3.21$\times$10^-8^ | 25.35 | 3.37$\times$10^-8^ |
| SHA-SnO_2_ | 14.32 | 22.59 | 8.66$\times$10^-8^ | 115.2 | 1.69$\times$10^-8^ |

**Table S4.** Summary of reported photovoltaic performance of perovskite solar cells with polymer treated SnO_2_.

| **Number** | **Polymer** | ***V_oc_ (V)*** | ***J_sc_***  ***(mA cm^-2^)*** | ***FF (%)*** | ***PCE (%)*** | **Year** | **Ref.** |
| --- | --- | --- | --- | --- | --- | --- | --- |
| 1 |   Polyethylene Glycol (PEG) | 1.12 | 22.67 | 81.9 | 22.67 | 2018 | [1] |
| 2 |   polyvinyl pyrrolidone (PVP) | 1.13 | 21.06 | 81 | 19.42 | 2018 | [2] |
| 3 | 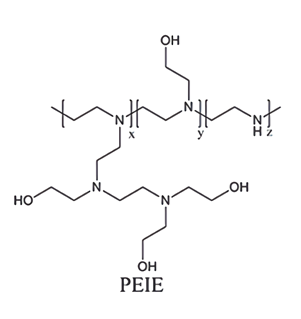  PEIE | 1.14 | 23.83 | 76 | 20.61 | 2019 | [3] |
| 4 | 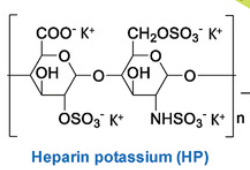  Heparin Potassium (HP) | 1.16 | 25.00 | 79.2 | 23.03 | 2020 | [4] |
| 5 |   Polyethylene Oxide-polypropylene Oxide-polyethylene Oxide (P123) | 1.16 | 24.21 | 77.1 | 21.69 | 2022 | [5] |
| 6 |   Poly(ethylene glycol) Diacrylate (PEGDA) | 1.14 | 25.24 | 81.00 | 23.31 | 2023 | [6] |
| 7 | 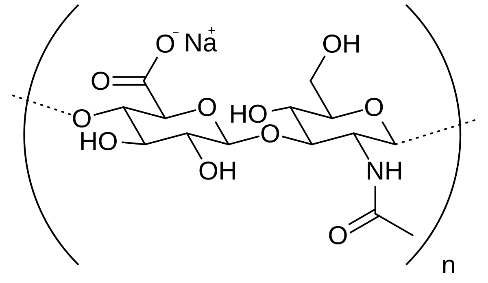  Soidum Hyaluronate (SHA) | 1.19 | 25.77 | 82.15 | 25.11 | 2025 | This work |

**Table S5.** Summary of reported photovoltaic performance of perovskite solar cells with additive engineering on SnO_2_ within the past five years.

| **Number** | **Additive** | ***V_oc_ (V)*** | ***J_sc_***  ***(mA cm^-2^)*** | ***FF (%)*** | ***PCE (%)*** | **Year** | **Ref.** |
| --- | --- | --- | --- | --- | --- | --- | --- |
| 1 |   Girard's Reagent T | 1.14 | 22.92 | 81.2 | 21.63 | 2021 | [7] |
| 2 |   Potassium Trifluoroacetate | 1.14 | 24.60 | 77.81 | 21.73 | 2022 | [8] |
| 3 |   2-Hydroxyethyl trimethylammonium chloride | 1.19 | 24.16 | 79.99 | 23.07 | 2022 | [9] |
| 4 |   Ammonium Formate | 1.15 | 23.44 | 82.89 | 22.37 | 2022 | [10] |
| 5 |   Formamidine Oxalate | 1.15 | 26.09 | 79.51 | 25.05 | 2023 | [11] |
| 6 |   Sodium Phytate | 1.16 | 25.06 | 81.43 | 23.77 | 2023 | [12] |
| 7 |   Potassium Citrate | 1.16 | 25.19 | 84.7 | 24.9 | 2023 | [13] |
| 8 |   Nitric Acid | 1.21 | 25.04 | 80.40 | 24.31 | 2024 | [14] |
| 9 |   Cyclohexyl-methylammonium Chloride | 1.18 | 25.55 | 84.19 | 25.47 | 2024 | [15] |
| 10 |   Citric Acid | 1.15 | 26.19 | 82.46 | 24.82 | 2024 | [16] |
| 11 |   Sodium Gluconate | 1.19 | 25.76 | 82.38 | 25.34 | 2024 | [17] |
| 12 |   Ectoine | 1.18 | 25.43 | 81.85 | 24.68 | 2025 | [18] |
| 13 |   Aminomethyl Phosphonic Acid | 1.14 | 26.46 | 80.29 | 24.22 | 2025 | [19] |

**References**

[1] Wei, J., Guo, F., Wang, X., Xu, K., Lei, M., Liang, Y., Zhao, Y., and Xu, D. (2018) SnO_2_-in-Polymer Matrix for High-Efficiency Perovskite Solar Cells with Improved Reproducibility and Stability. Advanced Materials, 30 (52), 1805153.

[2] Wang, D., Chen, S.C., and Zheng, Q. (2019) Poly(vinylpyrrolidone)-doped SnO_2_ as an Electron Transport Layer for Perovskite Solar Cells with Improved Performance. J Mater Chem C Mater, 7 (39), 12204–12210.

[3] Huang, X., Du, J., Guo, X., Lin, Z., Ma, J., Su, J., Feng, L., Zhang, C., Zhang, J., Chang, J., and Hao, Y. (2020) Polyelectrolyte-Doped SnO_2_ as a Tunable Electron Transport Layer for High-Efficiency and Stable Perovskite Solar Cells. Solar RRL, 4 (1), 1900336.

[4] You, S., Zeng, H., Ku, Z., Wang, X., Wang, Z., Rong, Y., Zhao, Y., Zheng, X., Luo, L., Li, L., Zhang, S., Li, M., Gao, X., and Li, X. (2020) Multifunctional Polymer-Regulated SnO_2_ Nanocrystals Enhance Interface Contact for Efficient and Stable Planar Perovskite Solar Cells. Advanced Materials, 32 (43), 2003990.

[5] Xu, Z., Zhou, X., Li, X., and Zhang, P. (2022) Polymer-Regulated SnO_2_ Composites Electron Transport Layer for High-Efficiency n–i–p Perovskite Solar Cells. Solar RRL, 6 (8), 2200092.

[6] Xiong, Z., Lan, L., Wang, Y., Lu, C., Qin, S., Chen, S., Zhou, L., Zhu, C., Li, S., Meng, L., Sun, K., and Li, Y. (2021) Multifunctional Polymer Framework Modified SnO_2_ Enabling a Photostable α-FAPbI_3_ Perovskite Solar Cell with Efficiency Exceeding 23%. ACS Energy Lett, 6 (11), 3824–3830.

[7] Bi, H., Zuo, X., Liu, B., He, D., Bai, L., Wang, W., Li, X., Xiao, Z., Sun, K., Song, Q., Zang, Z., and Chen, J. (2021) Multifunctional Organic Ammonium Salt-modified SnO_2_ Nanoparticles toward Efficient and Stable Planar Perovskite Solar Cells. J Mater Chem A Mater, 9 (7), 3940–3951.

[8] Xi, J., Yuan, J., Du, J., Yan, X., Tian, J., Xi, J., Yuan, J., Du, J., Tian, J., and Yan, X. (2022) Efficient Perovskite Solar Cells Based on Tin Oxide Nanocrystals with Difunctional Modification. Small, 18 (33), 2203519.

[9] Deng, J., Zhang, H., Wei, K., Xiao, Y., Zhang, C., Yang, L., Zhang, X., Wu, D., Yang, Y., and Zhang, J. (2022) Molecular Bridge Assisted Bifacial Defect Healing Enables Low Energy Loss for Efficient and Stable Perovskite Solar Cells. Adv Funct Mater, 32 (52), 2209516.

[10] Zheng, Z., Li, F., Gong, J., Ma, Y., Gu, J., Liu, X., Chen, S., and Liu, M. (2022) Pre-Buried Additive for Cross-Layer Modification in Flexible Perovskite Solar Cells with Efficiency Exceeding 22%. Advanced Materials, 34 (21), 2109879.

[11] Bian, L., Jia, Y., Zhao, Y., Dou, Z., Guo, Q., Duan, J., Dou, J., Sun, L., Zhang, Q., and Tang, Q. (2024) Target Therapy at Buried Interfaces toward Efficient and Stable Inorganic Perovskite Solar Cells. Chemical Engineering Journal, 496, 154189.

[12] Wang, S., Dai, R., Meng, X., Yang, J., and Chen, Y. (2024) A Chelating-Agent-Passivated Electron Transport Layer for Efficient Perovskite Solar Cells with Enhanced Reproducibility. Adv Funct Mater, 34 (13), 2310860.

[13] Dong, W., Zhu, C., Bai, C., Ma, Y., Lv, L., Zhao, J., Huang, F., Cheng, Y.B., and Zhong, J. (2023) Low-Cost Hydroxyacid Potassium Synergists as an Efficient In Situ Defect Passivator for High Performance Tin-Oxide-Based Perovskite Solar Cells. Angewandte Chemie International Edition, 62 (25), e202302507.

[14] Yun, H., Seo, Y., Seo, C., Kim, H.S., Yoo, S. Bin, Kang, B.J., Jeon, N.J., and Jung, E.H. (2024) Surface Engineering of Tin Oxide Nanoparticles by pH Modulation Facilitates Homogeneous Film Formation for Efficient Perovskite Solar Modules. Adv Energy Mater, 14 (25).

[15] Chen, P., Pan, W., Wang, S., Wang, Y., Yan, Z., Zheng, Q., Sun, W., Lan, Z., and Wu, J. (2024) Across-Interface Effect of Alkylamine Salt on the Formation of Intermediate Phase and Defect Passivation in High-Performance of Perovskite Solar Cells. Adv Funct Mater, 35 (2).

[16] Zhao, X., Qiu, Y., Wang, M., Wu, D., Yue, X., Yan, H., Fan, B., Du, S., Yang, Y., Yang, Y., Li, D., Cui, P., Huang, H., Li, Y., Park, N.G., and Li, M. (2024) Regulation of Buried Interface through the Rapid Removal of PbI_2_·DMSO Complex for Enhancing Light Stability of Perovskite Solar Cells. ACS Energy Lett, 9 (6), 2659–2669.

[17] Yuan, L., Zou, S., Zhang, K., Huang, P., Dong, Y., Wang, J., Fan, K., Lam, M.Y., Wu, X., Cheng, W., Tang, R., Chen, W., Liu, W., Wong, K.S., and Yan, K. (2024) Bottom Contact Engineering for Ambient Fabrication of >25% Durable Perovskite Solar Cells. Advanced Materials, 36 (41).

[18] Cheng, W., Huang, P., Gao, Z., Chen, Y., Ren, L., Feng, Q., Liu, X., Ahmad, S., and Zhou, Z. (2025) Molecular Bridging of Buried Interface Flattens Grain Boundary Grooves and Imparts Stress Relaxation for Performance Enhancement and UV Stability in Perovskite Solar Cells. Adv Energy Mater, 15 (30), 2501296.

[19] Gao, Y., Gong, W., Zhang, Z., Guo, J., Ma, J., Li, X., Zeng, Y., and Wu, M. (2025) Aminomethyl Phosphonic Acid as Highly Effective Multifunctional Additive for Modification of Electron Transport Layer and Perovskite in Photovoltaic Solar Cells. Angewandte Chemie - International Edition, 64 (23), e202424479.
